# Radiohybrid Ligands: A Novel Tracer Concept Exemplified by ^18^F- or ^68^Ga-Labeled rhPSMA Inhibitors

**DOI:** 10.2967/jnumed.119.234922

**Published:** 2020-05

**Authors:** Alexander Wurzer, Daniel Di Carlo, Alexander Schmidt, Roswitha Beck, Matthias Eiber, Markus Schwaiger, Hans-Jürgen Wester

**Affiliations:** 1 Chair of Pharmaceutical Radiochemistry, Technical University of Munich, Garching, Germany; and; 2 Department of Nuclear Medicine, Klinikum rechts der Isar, Technical University of Munich, Munich, Germany

**Keywords:** PSMA, ^18^F, prostate cancer, radiohybrid

## Abstract

When we critically assess the reason for the current dominance of ^68^Ga-labeled peptides and peptide-like ligands in radiopharmacy and nuclear medicine, we have to conclude that the major advantage of such radiopharmaceuticals is the apparent lack of suitable ^18^F-labeling technologies with proven clinical relevance. To prepare and to subsequently perform a clinical proof-of-concept study on the general suitability of silicon-fluoride-acceptor (SiFA)–conjugated radiopharmaceuticals, we developed inhibitors of the prostate-specific membrane antigen (PSMA) that are labeled by isotopic exchange (IE). To compensate for the pronounced lipophilicity of the SiFA unit, we used metal chelates, conjugated in close proximity to SiFA. Six different radiohybrid PSMA ligands (rhPSMA ligands) were evaluated and compared with the commonly used ^18^F-PSMA inhibitors ^18^F-DCFPyL and ^18^F-PSMA-1007. **Methods:** All inhibitors were synthesized by solid-phase peptide synthesis. Human serum albumin binding was measured by affinity high-performance liquid chromatography, whereas the lipophilicity of each tracer was determined by the *n*-octanol/buffer method. In vitro studies (IC_50_, internalization) were performed on LNCaP cells. Biodistribution studies were conducted on LNCaP tumor–bearing male CB-17 SCID mice. **Results:** On the laboratory scale (starting activities, 0.2–9.0 GBq), labeling of ^18^F-rhPSMA-5 to -10 by IE was completed in < 20 min (radiochemical yields, 58% ± 9%; radiochemical purity, >97%) with molar activities of 12–60 GBq/μmol. All rhPSMAs showed low nanomolar affinity and high internalization by PSMA-expressing cells when compared with the reference radiopharmaceuticals, medium-to-low lipophilicity, and high human serum albumin binding. Biodistribution studies in LNCaP tumor–bearing mice revealed high tumor uptake, sufficiently fast clearance kinetics from blood, low hepatobiliary excretion, fast renal excretion, and very low uptake of ^18^F activity in bone. **Conclusion:** The novel ^18^F-rhPSMA radiopharmaceuticals developed under the radiohybrid concept show equal or better targeting characteristics than the established ^18^F-PSMA tracers ^18^F-DCFPyL and ^18^F-PSMA-1007. The unparalleled simplicity of production, the possibility to produce the identical ^68^Ga-labeled ^19^F-^68^Ga-rhPSMA tracers, and the possibility to extend this concept to true theranostic radiohybrid radiopharmaceuticals, such as F-Lu-rhPSMA, are unique features of these radiopharmaceuticals.

Since the clinical introduction of ^68^Ga-labeled somatostatin receptor ligands in the first decade of this century, ^68^Ga has gained increased interest and importance. As a consequence, more and more peptidic radiopharmaceuticals have been developed and assessed, whereupon approved ^68^Ge/^68^Ga generators have become commercially available. Thus, fostered by the success of the first ^68^Ga radiopharmaceuticals, such as the approved ^68^Ga-labeling kits NETSPOT (kit for the preparation of ^68^Ga-DOTATATE) and SOMAKIT TOC (kit for the preparation of ^68^Ga-DOTATOC), a unique ^68^Ge/^68^Ga generator–based radiopharmacy concept has been established in parallel to the cyclotron-based production of radiopharmaceuticals (1,2). Although “fast and inexpensive production” and “ease of generator-based syntheses” are widely accepted unique features of this concept, it has to be noted that these assessments are based on a comparison with the current clinically established state-of-the-art ^18^F-labeling technologies.

When we critically assess the reason for the current relevance of ^68^Ga in radiopharmacy and nuclear medicine we have to conclude that the apparent lack of suitable ^18^F-labeling technologies with proven clinical relevance is the major advantage for ^68^Ga-labeled peptides and peptide-like radiopharmaceuticals.

Since ^68^Ga labeling by complexation is fast and efficient, none of the current clinically established ^18^F-labeling technologies can offer comparable levels of simplicity and speed (3–5). To overcome these limitations, a variety of alternative ^18^F-labeling techniques have been investigated and assessed, and the range of ^18^F labeling has been extended from C-^18^F bond formation to the formation of ^18^F-bonds with silicon (6), boron (7), and aluminum (8).

The latter relies on the strong chemical bond between aluminum and fluoride, which is exploited for complexation of ^18^F-AlF^2+^, especially by suitable NOTA- (1,4,7-triazacyclononane-1,4,7-triacetic acid-) conjugated ligands (8,9). In the recent publication by Liu et al., a new NOTA derivative of PSMA-617, Al^18^F-PSMA-BCH, is described (10). Its production is performed in a formal 2 step-procedure, consisting of the formation of ^18^F-AlF^2+^ (5 min at ambient temperature [r.t.]) and subsequent complexation by means of 80 nmol precursor at 110°C for 15 min and purification by a simple solid-phase extraction (SPE) process. Manual syntheses yielded the product in 32% ± 5% radiochemical yield (RCY) and 99% radiochemical purity (RCP) (10). Further elegant labeling approaches based on boron compounds were introduced by Perrin et al. (11,12). Herein, arylfluoroborates were applied for synthesis of PET probes by means of ^18^F nucleophilic substitution of borate esters or ^19^F-^18^F isotopic exchange (IE) of organotrifluoroborates (7,11–13). In a recent paper by Kuo et al. on 8 different trifluoroborate-conjugated PSMA inhibitors, the labeling started with about 37 GBq and resulted in 4%–16% yield (uncorrected) when using 100 nmol precursor (12). To obtain high RCP (>99%), HPLC (high-performance liquid chromatography) purification was necessary; when HPLC purification was replaced by SPE, RCP dropped to >95%. The use of a precursor amount of 1,000 nmol was exemplarily investigated on one compound, resulting in 36% yield (4% yield on the 100 nmol level, both yields uncorrected) (12). Another recently developed methodology allows for chemoselective transition-metal-assisted ^18^F-deoxyfluorination of a tyrosine residue in small peptides (14). ^18^F-deoxyfluorination of a series of small peptides was performed using 5 μmol peptide precursor (corresponding to about 5–7.5 mg of typically used peptides of 1,000–1,500 g/mol) and a final HPLC purification within an overall synthesis time of 80–100 min. The authors described that reduction of the peptide amount to 1.5 μmol (1.5–2.3 mg) is possible, leading to a reduction of the RCY by about 50%, which corresponds to 10%–20% RCY for the peptides used or 5%–10% uncorrected yield 80–100 min after end-of-bombardment (14).

Regarding the Si-^18^F bond formation, initial experiments were performed with SiF_4_ and alkyfluorosilanes in 1958 (15–17). In 2006, Schirrmacher et al. proved that sterically demanding substituents around the silicon (e.g., phenyls or branched alkyls) could preserve the Si-^18^F bond and prevent fast hydrolysis in aqueous medium (6). These results were supported by Blower et al. on ^18^F-fluorination of alkoxysilanes in nucleophilic substitutions (18) and a systematic evaluation of different silicon-fluoride-acceptor (SiFA) building blocks by Höhne et al. (19). A kinetic analysis for the isoenergetic replacement of ^19^F by the PET-isotope ^18^F in a SiFA moiety (20) revealed a low-energy barrier of only 15.7 kcal/mol, which explains the fast ^18^F-for-^19^F IE reaction at r.t. within 5 min, yielding ^18^F-SiFA–conjugated tracers in high yields (>40%) and high molar activities (>60 GBq/μmol). Moreover, the absence of side products allows for a simple cartridge-based purification, resulting in a total synthesis time of <30 min (21–23).

In vivo studies in mice with ^18^F-SiFA-TATE, an octreotate-based somatostatin receptor agonist, revealed no elevated activity accumulation in bone and thus high hydrolytic stability of the Si-^18^F bond (21,24). However, due to the bulky and highly lipophilic SiFA, the activity was predominantly accumulating in the liver and gastrointestinal system (21,24). With the aim to increase hydrophilicity, incorporation of hydrophilic modifiers, such as carboxylic acids, carbohydrates, polyethylene glycol, and combinations thereof, were tested (21,22,25). Moreover, a positive charge was introduced in the SiFA-building block (20,21). Despite the recent efforts to decrease the lipophilicity, none of the SiFA-bearing ligands described so far showed the potential for first proof-of-concept studies in men also necessary to confirm sufficient hydrolytic stability of the Si-^18^F-bond in men.

To design SiFA-based prostate-specific membrane antigen (PSMA) inhibitors with sufficient hydrophilicity, we developed and investigated compounds that combine a SiFA moiety and a chelator (or chelate) in a single molecule, named radiohybrid PSMA inhibitors (rhPSMAs). Such rhPSMA ligands can be labeled with ^18^F by IE, whereas the chelator is used for complexation of a cold metal (e.g., ^nat^Ga or ^nat^Lu), or can be labeled with a radiometal (e.g., ^68^Ga, ^177^Lu, or ^225^Ac), whereas the SiFA moiety is nonradioactive (Fig. 1). The new series of tracers was evaluated in vitro (IC_50_, binding to and internalization into LNCaP cells, binding to human serum albumin [HSA]) and in vivo (LNCaP tumor–bearing severe combined immunodeficiency [SCID] mice) and compared with the best recently described ^18^F-labeled PSMA inhibitors DCFPyL and PSMA-1007 (26,27).

**FIGURE 1.**
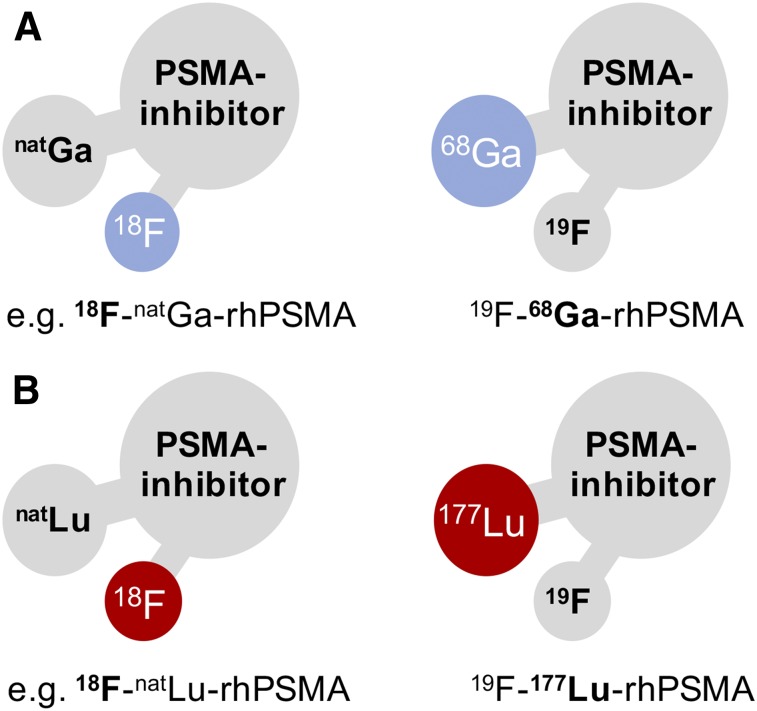
Radiohybrid concept exemplified on PSMA inhibitors: a molecular species that offers 2 binding sites for radionuclides, here a SiFA for ^18^F and a chelator for radiometallation. One of these binding sites is “labeled” with a radioisotope, the other one is silent, thus “labeled” with a nonradioactive isotope. These pair of compounds, either pure imaging pairs (A) or theranostic pairs (B) represent chemically identical species (monozygotic chemical twins) and thus exhibit identical in vivo characteristics (e.g., affinity, lipophilicity, pharmacokinetics). ^68^Ga in A and ^177^Lu in B are examples that can be substituted by other radiometals.

## MATERIALS AND METHODS

### General Information

The Fmoc-(9-fluorenylmethoxycarbonyl-) and all other protected amino acid analogs were purchased from Bachem or Iris Biotech. The tritylchloride polystyrene resin was obtained from PepChem. Chematech delivered the chelators DOTAGA (2-(4,7,10-tris(carboxymethyl)-1,4,7,10-tetraazacyclododecan-1-yl)pentanedioic acid), DOTA (1,4,7,10-tetraazacyclododecane-1,4,7,10-tetraacetic acid), NOTA, and derivatives thereof. All necessary solvents and other organic reagents were purchased from either Alfa Aesar, Sigma-Aldrich, Fluorochem, or VWR.

The tBu-protected PSMA-addressing binding motifs Lys-urea-Glu ((tBuO)KuE(OtBu)_2_) and Glu-urea-Glu ((tBuO)EuE(OtBu)_2_) as well as the derivative PfpO-Sub-(tBuO)KuE(OtBu)_2_ (Pentafluorophenyl-suberic acid active ester of the tBu-protected EuK binding motif) were prepared in analogy to previously described procedures (28–30). Syntheses of the silicon-fluoride-acceptor 4-(di-tert-butylfluorosilyl)benzoic acid (SiFA-BA) and the alkyne-functionalized TRAP chelator (1,4,7-triazacyclononane-1,4,7-tris[methyl(2-carboxyethyl)phosphinic acid) were performed according to the literature protocols (31,32).

Solid-phase synthesis of the peptides was carried out by manual operation using a syringe shaker (Intelli, Neolab). Analytical and preparative HPLC were performed using Shimadzu gradient systems (Shimadzu), each equipped with a SPD-20A UV/Vis detector (220 nm, 254 nm). A Nucleosil 100 C18 (125 × 4.6 mm, 5 μm particle size) column (CS Chromatographie Service) was used for analytical measurements at a flow rate of 1 mL/min. Both specific gradients and the corresponding retention times are cited in the text. Preparative HPLC purification was done with a Multospher 100 RP 18 (250 × 10 mm, 5 μm particle size) column (CS Chromatographie Service) at a constant flow rate of 5 mL/min. Analytical and preparative radio HPLC was performed using a Nucleosil 100 C18 (5 μm, 125 × 4.0 mm) column (CS Chromatographie Service). Eluents for all HPLC operations were water (solvent A) and acetonitrile (solvent B), both containing 0.1% trifluoroacetic acid. Radioactivity was detected through connection of the outlet of the UV-photometer to a HERM LB 500 NaI detector (Berthold Technologies). Electrospray ionization-mass spectra for characterization of the substances were acquired on an expression LCMS mass spectrometer (Advion, Harlow). Nuclear magnetic resonance spectra were recorded on Bruker (Billerica, USA) AVHD-300 or AVHD-400 spectrometers at 300 K. Activity quantification was performed using a 2480 WIZARD2 automatic gamma counter (PerkinElmer). Radio–thin-layer chromatography was carried out with a Scan-RAM detector (LabLogic Systems).

### Chemical Synthesis

**FIGURE 2.**
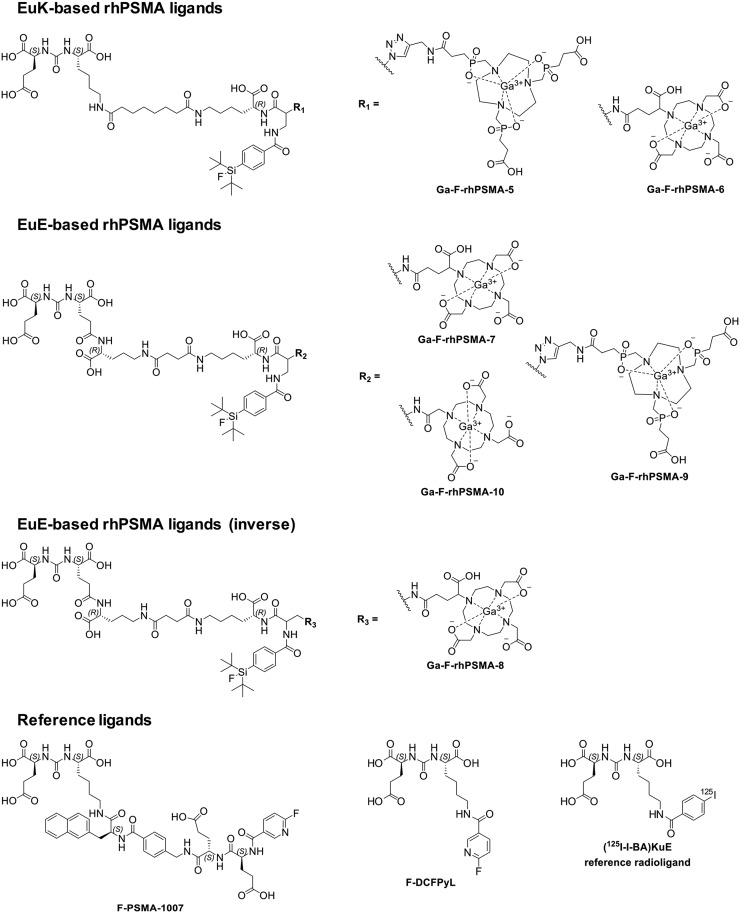
Radiohybrid (rh) PSMA ligands comprising the KuE- or EuE-based PSMA inhibition motif, a SiFA moiety and a TRAP-, DOTA-, or DOTAGA-chelator. For comparative evaluations, the well-established PSMA-addressing ligands F-DCFPyL and F-PSMA-1007 were used (26,27). The reference radioligand for in vitro determinations was (^125^I-I-BA)KuE (28).

The rhPSMA ligands were prepared via a mixed solid-phase/solution-phase synthetic strategy. Final purification of the compounds was achieved by reversed-phase HPLC. A detailed description of the synthesis of uncomplexed rhPSMA-5 to -10 (Supplemental Fig. 1 to Supplemental Fig. 12; supplemental materials are available at http://jnm.snmjournals.org), including cold gallium complexation and their characterization is provided in the supplemental information. Structural formulas of rhPSMAs and of the reference ligands, ^19^F-DCFPyL^, 19^F-PSMA-1007 and (((*S*)-1-carboxy-5-(4-(^125^I-iodo)benzamido)pentyl)carbamoyl)-*L*-glutamic acid ((^125^I-I-BA)KuE) are depicted in Figure 2 (26–28).

### Radiolabeling

#### Automated ^68^Ga Labeling

^68^Ga labeling was performed using an automated system (GallElut^+^ by Scintomics) as described previously (33).

#### Manual ^18^F Labeling

^18^F-fluoride (∼0.6–2.0 GBq/mL) was provided by the Klinikum rechts der Isar. For manual ^18^F labeling, a previously published procedure was slightly modified (23). Briefly, aqueous ^18^F^−^ was passed through a strong anion exchange cartridge (Sep-Pak Accell Plus QMA Carbonate Plus Light cartridge, 46 mg, 40 μm; Waters), which was preconditioned with 10 mL of water. Most of the remaining water was removed with 20 mL of air, and any residual was removed by rinsing the cartridge with 10 mL of anhydrous acetonitrile (for DNA synthesis, VWR) followed by 20 mL of air. For cartridge elution, [K^+^⊂2.2.2]OH^−^ kits, containing a lyophilized mixture of 2.2.2-cryptand (Kryptofix 222, 110 μmol, 1.1 eq., Sigma Aldrich) and KOH (100 μmol, 1.0 eq., 99.99% semiconductor grade, Sigma Aldrich) were used, which were dissolved in 500 μL of anhydrous acetonitrile before the elution process. The eluate was then partly neutralized with 30 μmol of oxalic acid (99.999%, trace metals basis, Sigma Aldrich) in anhydrous acetonitrile (1 M, 30 μL). The resulting mixture was used as a whole or aliquot for fluorination of 10–150 nmol of a respective labeling precursor 1 mM in anhydrous dimethyl sulfoxide (>99.9%, Sigma Aldrich) for 5 min at r.t. For purification of the tracer, an Oasis HLB Plus Light cartridge (30 mg sorbent, 30 μm particle size; Waters), preconditioned with 10 mL of water, was used. The labeling mixture was diluted with 9 mL phosphate-buffered saline (PBS, pH 3, adjusted with 1 M aqueous HCl) and passed through the cartridge followed by 10 mL PBS (pH 3) and 10 mL air. The peptide was eluted with 0.3–2.0 mL of a 1:1 mixture (v/v) of ethanol in water. RCP of the ^18^F-labeled compound was determined by radio–thin-layer chromatography (Silica gel 60 RP-^18^F_254_s, mobile phase: 3:2 mixture (v/v) of acetonitrile in water supplemented with 10% of 2 M sodium acetate solution and 1% of trifluoroacetic acid) and radio RP-HPLC (Nucleosil 100 C18, 5 μm, 125 × 4.0 mm, mobile phases water and acetonitrile, both containing 0.1% trifluoroacetic acid (see supporting information).

#### Lipophilicity and Binding to HSA

Approximately 1 MBq of the labeled tracer was dissolved in 1 mL of a 1:1 mixture (v/v) of PBS (pH 7.4) and *n*-octanol in a reaction vial (*n* = 6). After vigorous mixing of the suspension for 3 min at r.t., the vial was centrifuged at 15,000*g* for 3 min (Biofuge 15, Heraus Sepatech), and 100 μL aliquots of both layers were measured in a γ-counter.

HSA binding of the rhPSMA ligands was determined according to a previously published procedure via HPLC, using a Chiralpak HSA column (50 × 3 mm, 5 μm, H13H-2433, Daicel) with minor modifications (34).

### In Vitro Experiments

#### Cell Culture

PSMA-positive LNCaP cells (300265; Cell Lines Service) were cultivated in Dulbecco modified Eagle medium (DMEM)/Nutrition Mixture F-12 with GlutaMAX (1:1, DMEM-F12, Biochrom) supplemented with fetal bovine serum (10%, FBS Zellkultur) and kept at 37°C in a humidified CO_2_ atmosphere (5%). A mixture of trypsin and ethylenediaminetetraacetic acid (0.05%, 0.02%) in PBS (Biochrom) was used to harvest cells. Cells were counted with a Neubauer hemocytometer (Paul Marienfeld).

#### Affinity Determinations (IC_50_) and Internalization Studies

Competitive binding studies were determined on LNCaP cells (1.5 × 10^5^ cells in 1 mL/well) after incubation at 4°C for 1 h, using (^125^I-I-BA)KuE (0.2 nM/well) as reference radioligand (*n* = 3). Internalization studies of the radiolabeled ligands (0.5 nM/well) were performed on LNCaP cells (1.25 × 10^5^ cells in 1 mL/well) at 37°C for 1 h and accompanied by (^125^I-I-BA)KuE (0.2 nM/well), as reference ligand. Data were corrected for nonspecific binding and normalized to the specific-internalization observed for the radioiodinated reference compound (*n* = 3).

### In Vivo Experiments

All animal experiments were conducted in accordance with general animal welfare regulations in Germany (German animal protection act, as amended on May 18, 2018, Art. 141 G v. 29.3.2017 I 626, approval no. 55.2-1-54-2532-71-13) and the institutional guidelines for the care and use of animals. To establish tumor xenografts, LNCaP cells (∼10^7^ cells) were suspended in 200 μL of a 1:1 mixture (v/v) of DMEM F-12 and Matrigel (BD Biosciences, Germany) and inoculated subcutaneously onto the right shoulder of 6- to 8-wk-old CB17-SCID mice (Charles River). Mice were used for experiments when tumors had grown to a diameter of 5–10 mm (3–6 wk after inoculation).

#### Biodistribution

Approximately 2–20 MBq (0.2 nmol) of the radioactive-labeled PSMA inhibitors were injected into the tail vein of LNCaP tumor–bearing male CB-17 SCID mice that were sacrificed at 1 h after injection (*n* = 3 for ^68^Ga-^19^F-rhPSMA-7 to -9 and ^18^F-rhPSMA-7, *n* = 4 for ^68^Ga-^19^F-rhPSMA-10, ^18^F-DCFPyL and ^18^F-PSMA-1007). Selected organs were removed, weighed, and measured in a γ-counter.

## RESULTS

### Synthesis and Radiolabeling

Synthesis of uncomplexed rhPSMA-5 to -10 was performed via a straightforward mixed solid-phase/solution phase-synthetic strategy (supplemental data).

Final products were obtained in a chemical purity of greater than 97%, determined by HPLC (220 nm). Cold metal complexation with a molar excess of Ga(NO_3_)_3_:1.5-fold molar excess for TRAP-based conjugates, 3.0-fold molar excess for DOTA-based conjugates led to a quantitative formation of the respective ^nat^Ga-rhPSMA ligand (Fig. 2).

^68^Ga labeling of uncomplexed rhPSMA was performed in a standard automated procedure in RCYs of 60% ± 7% and molar activities of 59 ± 20 GBq/μmol. RCPs were more than 97% for all compounds.

^18^F labeling was performed by a ^19^F/^18^F IE reaction already described for SiFA compounds in a manual procedure (23). Drying of aqueous ^18^F-fluoride was performed through ^18^F-fixation on a strong anion exchange cartridge (QMA, Waters), followed by removal of water with air and anhydrous acetonitrile, according to the previously described Munich Method (35). Dried ^18^F-fluoride was eluted from the QMA by [K^+^2.2.2]OH^−^ directly into a mixture of the labeling precursor and oxalic acid in 150 μL of dimethyl sulfoxide and 30 μL of MeCN (recovery of ^18^F-fluoride > 95%). The IE reaction was completed in 5 min at r.t. Due to the chemical identity of the starting material and radiolabeled product and the absence of chemical side products, a cartridge-based purification yielded the purified ligand in a total synthesis time of approximately 20 min in an RCP of more than 97%. The ^18^F-rhPSMA ligands could be obtained in RCYs of 58% ± 9% (*n* = 11, 50–150 nmol precursor) and molar activities of 12–60 GBq/μmol, when using starting activities of 0.2–9.0 GBq (exemplary HPLC analysis is shown in Supplemental Figure 13).

### In Vitro Characterization

**FIGURE 3.**
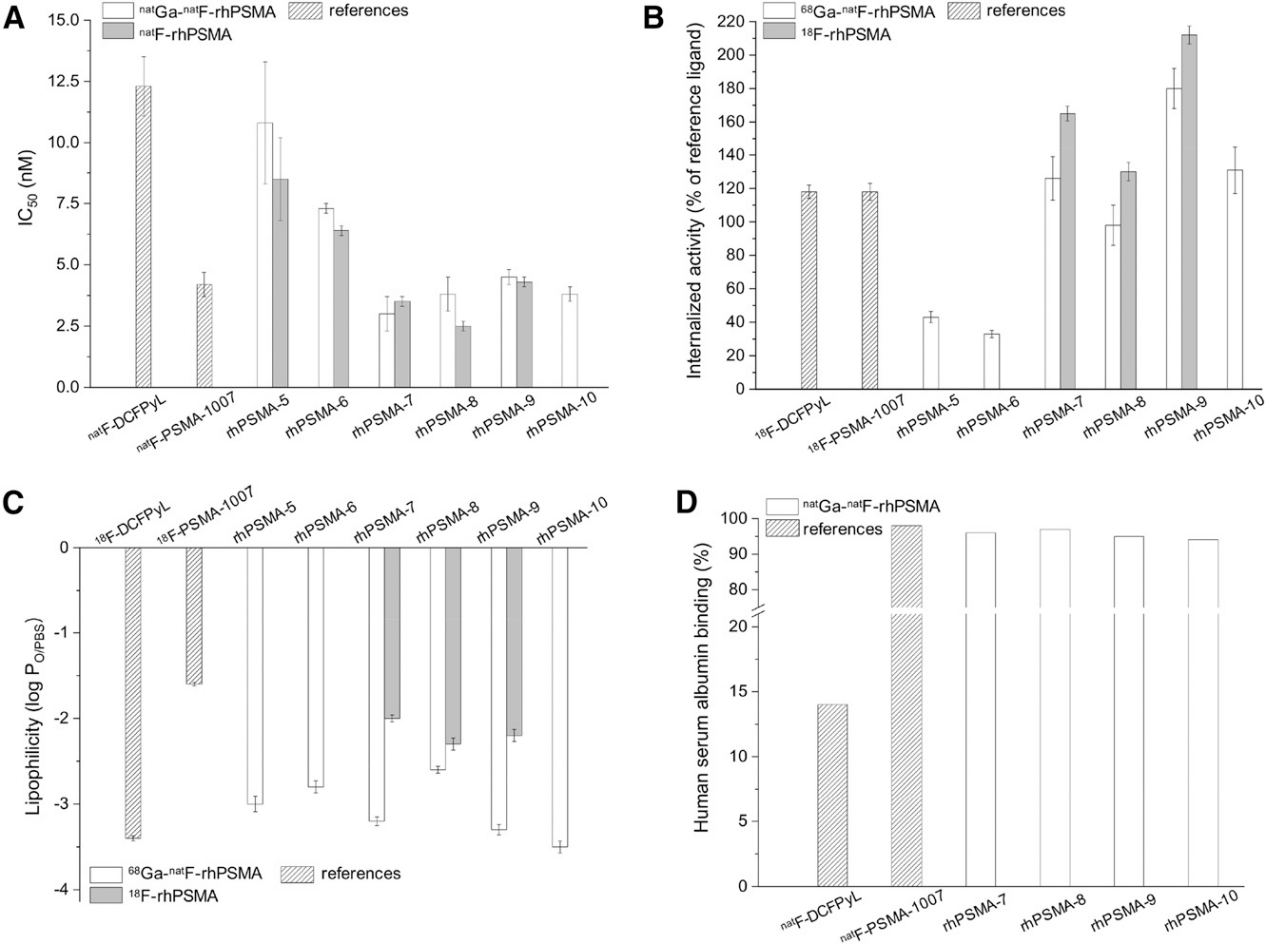
(A) Binding affinities (IC_50_ in nM, 1 h, 4°C; *n* = 3) of ^nat^Ga-^19^F-rhPSMA-5–10 (white bars), ^19^F-rhPSMA-5–10 with free chelator (gray bars), and ^19^F-DCFPyL and ^19^F-PSMA-1007 (references). (B) Internalized activity of ^18^F-DCFPyL, ^18^F-PSMA-1007, and ^68^Ga-^19^F-rhPSMA-5–10 (white bars) and ^18^F-rhPSMA-5–10 with free chelator (gray bars), in LNCaP cells (1 h, 37°C) as percentage of the reference ligand (^125^I-I-BA)KuE (*n* = 3). (C) Lipophilicity of ^18^F-DCFPyL, ^18^F-PSMA-1007, and ^68^Ga-^19^F-rhPSMA-5–10 (white bars) and ^18^F-rhPSMA-5–10 with free chelator (gray bars), expressed as *n*-octanol/PBS (pH 7.4) partition-coefficient (log P_oct/PBS_; *n* = 6). (D) HSA binding of ^19^F-DCFPyL, ^19^F-PSMA-1007, and ^nat^Ga-^19^F-rhPSMA-5–10 (white bars), determined on a Chiralpak HSA column. Data of reference ligands ^18/19^F-DCFPyL and ^18/19^F-PSMA-1007 were taken from a previously published study (30). Values are expressed as mean ± SD.

In vitro data of the synthesized (radio)metal-chelated rhPMSA ligands are summarized in Figure 3 and Supplemental Table 1; data from the well-established fluorinated PSMA ligands DCFPyL and PSMA-1007, evaluated under the same experimental conditions, were taken from a previously published study by our group and are included for comparison (26,27,30). Due to the chemical identity of the ^68^Ga-^19^F-rhPSMA with the respective ^nat^Ga-^18^F-rhPSMA compound, only the ^68^Ga-labeled twin was evaluated in experiments that required a radioactive compound. Moreover, the uncomplexed ^18^F-labeled rhPSMA ligands were tested to assess the influence of the chelated metal cation on the in vitro behavior.

The PSMA-binding affinities (IC_50_) (Fig. 3A) were determined in a competitive binding assay on LNCaP human prostate carcinoma cells, using (^125^I-I-BA)KuE as radioligand. rhPSMA-5 and 6, which are based on the Lys-urea-Glu (KuE) scaffold, showed PSMA affinities somewhat better than that obtained for ^19^F-DCFPyL. Higher PSMA affinities were measured for the reference ligand ^19^F-PSMA-1007 and the Glu-urea-Glu-(EuE-)-based inhibitors rhPSMA-7 to rhPSMA-10. For the individual rhPSMA inhibitors in their Ga-complexed and metal-free forms, similar IC_50_s were found.

The extent of internalization was determined for each ^68^Ga-^19^F-rhPSMA compound and uncomplexed ^18^F-rhPSMA-7 to -9 on LNCaP cells (1 h, 37°C) and normalized to the specific internalization of the reference radioligand (^125^I-I-BA)KuE, which was assayed in a parallel experiment for each study (Fig. 3B). Compared with the KuE-based rhPSMAs and corresponding with the trend observed in the affinity studies, internalization was considerably higher for all EuE-motif–based rhPSMA inhibitors. Especially, the uncomplexed ^18^F-fluorinated rhPSMA ligands displayed higher internalization rates as determined for the Ga-chelated analogs and also the reference ligands ^18^F-DCFPyL and ^18^F-PSMA-1007.

For all newly developed rhPSMA inhibitors, partition-coefficients (log P_oct/PBS_, pH 7.4) between −2.0 and −3.5 were determined (Fig. 3C). Interestingly, unchelated ^18^F-labeled compounds, when compared with the Ga-complexed counterparts, exhibited higher lipophilicity. A similar high hydrophilicity was determined for ^18^F-DCFPyL (−3.4), whereas ^18^F-PSMA-1007 was found to be of rather lipophilic nature (−1.6).

Binding to HSA was assessed by means of a recently described HPLC method (Fig. 3D) (34). Despite their high hydrophilicity, all SiFA-containing ligands exhibited strong HSA, interactions with binding more than 94% (^19^F-PSMA-1007 and ^19^F-DCFPyL: 98% and 14% HSA binding, respectively).

### In Vivo Characterization

**FIGURE 4.**
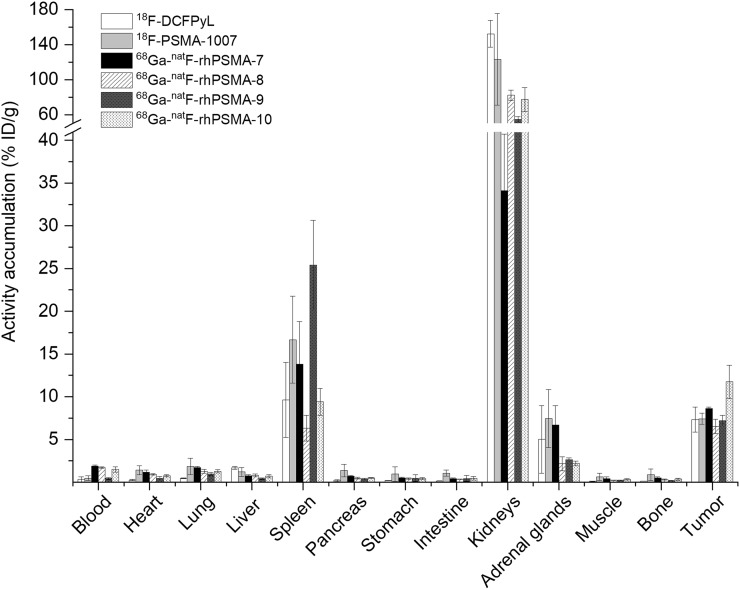
Biodistribution of ^68^Ga-^19^F-rhPSMA-7 to -10 and the reference ligands ^18^F-DCFPyL and ^18^F-PSMA-1007 at 1 h after injection in LNCaP tumor–bearing SCID mice (*n* = 3 for ^68^Ga-^19^F-rhPSMA-7 to -9, *n* = 4 for ^68^Ga-^19^F-rhPSMA-10, ^18^F-DCFPyL, and ^18^F-PSMA-1007). Data for reference ligands were taken from a previously published study by our group (30). Values are expressed as a percentage injected dose per gram (%ID/g), mean ± SD.

Taking into account the results of the in vitro assessment, only the EuE-based ligands ^68^Ga-^19^F-rhPSMA-7 to -10 were evaluated in biodistribution studies in male LNCaP tumor–bearing CB17 SCID mice at 1 h after injection and compared with the biodistribution of ^18^F-DCFPyL and ^18^F-PSMA-1007 (Fig. 4 and Supplemental Table 2) (30).

The comparative biodistribution study revealed that all of the examined ligands displayed similar pharmacokinetics with high uptake in PSMA-expressing tissue, for example, LNCaP tumors and kidneys, and in the spleen and adrenal gland. Compared with the fluorinated reference ligands, tumor uptake at 1 h after injection was similar for ^18^F-DCFPyL, ^18^F-PSMA-1007, and ^68^Ga-^19^F-rhPSMA-7, -8, and -9 and somewhat higher for ^68^Ga-^19^F-rhPSMA-10. Nontarget accumulation was low for all tracers with fast clearance via the renal pathway, except for ^18^F-PSMA-1007, which showed higher uptake in a variety of organs, such as the gastrointestinal system, but also lung and pancreas. Compared with all other tracers, ^18^F-DCPFyL and ^68^Ga-^19^F-rhPSMA-9 were more rapidly cleared from the blood within 1 h after injection.

### Biodistribution of ^68^Ga-^19^F-rhPSMA-7 and ^18^F-rhPSMA-7

**FIGURE 5.**
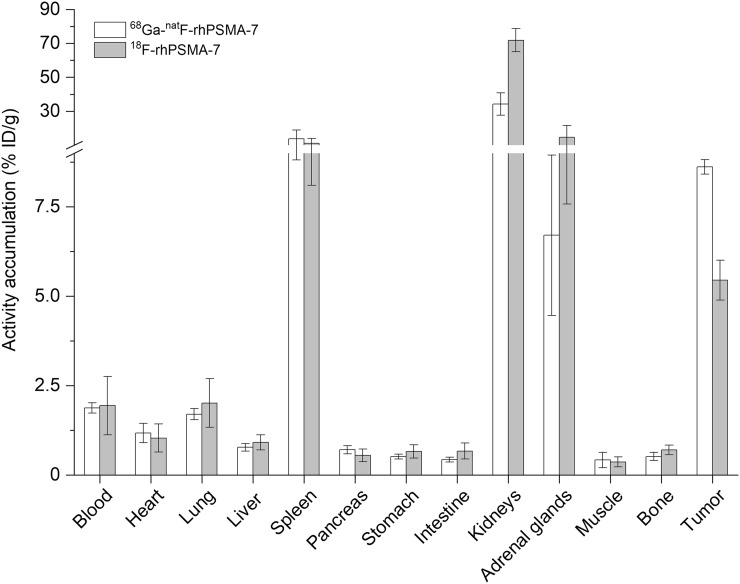
Comparative biodistribution of ^68^Ga-^19^F-rhPSMA-7 (white bars) and ^18^F-rhPSMA-7 (gray bars) at 1 h after injection in LNCaP tumor–bearing SCID mice (*n* = 3). Data are expressed as percentage injected dose per gram (%ID/g) (mean ± SD).

The biodistributions of uncomplexed ^18^F-rhPSMA-7 and ^68^Ga-labeled ^19^F-rhPSMA-7 were compared to examine the influence of the presence of the free chelator and a radiometal chelate on the in vivo behavior in male, LNCaP tumor–bearing SCID mice at 1 h after injection (Fig. 5 and Supplemental Table 2).

The uptake profiles of ^68^Ga-^19^F-rhPSMA-7 and unmetalated ^18^F-rhPSMA-7 in mice were found to be identical, with similar low uptake in most organs and pronounced uptake in the spleen, kidneys, adrenal gland, and tumor tissue. Although marked differences were found in the kidneys, in which the uncomplexed ^18^F-labeled ligand displayed stronger accumulation (72 vs. 34 percentage injected dose per gram), it remains questionable whether this difference is representative for the application in men. Interestingly, when compared with uncomplexed ^18^F-rhPSMA-7, a 1.6-fold higher tumor uptake was found for ^68^Ga-^19^F-rhPSMA-7. Again, no elevated bone accumulation was found for ^18^F-rhPSMA-7, indicating the absence of free ^18^F-fluoride.

## DISCUSSION

With the aim to develop a ^18^F-labeled PSMA-targeted inhibitor with excellent labeling and thus production properties, we combined for the first time, to our knowledge, a chelator and a SiFA moiety in a single inhibitor. Although the initial premise of this concept was driven by the expectation that a chelator (or a chelate) will significantly improve the hydrophilicity of the resulting SiFA-based tracer, several additional advantages of this radiohybrid concept became apparent. First, both the SiFA and the chelator can be labeled in an independent manner using the unprotected precursor, resulting in either ^18^F-M-rhPSMA (M = metal) or ^19^F-R-rhPSMA (R = radiometal), the latter to be used for imaging (e.g., ^68^Ga for PET, ^111^In for SPECT), or for radioligand therapy (e.g., ^177^Lu). The corresponding radiopharmaceuticals, for example, ^18^F-^nat^Ga-rhPSMA and ^19^F-^68^Ga-rhPSMA, are chemically identical molecules. Thus, they represent monozygotic chemical twins that should result in almost identical PET scans, with only slight differences determined by the nuclear properties of the chosen radioisotope. In addition, when using ^18^F in combination with a therapeutic radioisotope, such as ^177^Lu, the resulting twins, ^18^F-^nat^Lu-rhPSMA or ^19^F-^177^Lu-rhPSMA, could for the first time truly bridge ^18^F PET and radioligand therapy. Although speculative, such tracers might be interesting tools for pretherapeutic patient stratification, pretherapeutic dosimetry, and radioligand therapy with a single tracer by exploiting ^18^F and the most suitable therapeutic radioisotope (if also available as nonradioactive isotope).

Thus, and with great enthusiasm, we developed and evaluated a series of PSMA-targeted radiohybrid inhibitors. By slightly modifying the ^18^F-labeling procedure for IE on SiFA moieties (23), ^18^F-rhPSMA ligands could be obtained in manual laboratory experiments in up to 58% RCY, with molar activities of up to 60 GBq/μmol, similar to those reported in previous works of SiFA-bearing compounds (21–24). The combination of the Munich Drying Method, which comprises a simple and fast drying of aqueous ^18^F-fluoride on a solid phase cartridge and the subsequent elution of dry ^18^F-fluoride (35); the rapid and efficient ^18^F-for-^19^F IE at r.t.; and the possibility to purify the final product by solid-phase extraction resulted in a fast, but still not optimized, nonautomated production that was completed in less than 20 min, with an RCY of about 55% (not optimized) and RCP of more than 97%.

In the context of SiFA-conjugated ligands, we were able to overcome the previously unresolved lipophilicity problem. Even the incorporation of more or less complex combinations of hydrophilic auxiliaries could not compensate for the pronounced lipophilic influence of the SiFA-group (log P SiFA-lin-TATE = −1.21 (21); log P Ga-DOTATATE = −3.69 (36)) and the associated unsuitable biodistribution of such conjugates, a main obstacle for proof-of-concept studies in men. As described here, a chelator or a related metal chelate, conjugated in close proximity to a SiFA moiety of a rhPSMA ligand, increases the overall hydrophilicity of the inhibitor, whereas SiFA as lipophilic moiety and HSA binder decelerates blood clearance kinetics and avoids rapid and extensive occurrence of activity in the bladder. All rhPSMAs showed log *P* values between −2.0 and −3.5 (Fig. 3C) and thus exceeded the hitherto lowest lipophilicity of a SiFA-based ligand described in the literature, an α_v_β_3_ integrin–binding RGD-peptide with a log P of −2.0 (22). Interestingly, the Ga-chelated rhPSMA ligands displayed a higher hydrophilicity, compared with the respective uncomplexed analogs, even though their carboxylates are coordinated to the metal ion. Whether this unexpected observation is a general characteristic of rhPSMA is still under investigation.

Less surprising, and when compared with the KuE-based inhibitors rhPSMA-5 and -6, the EuE-based rhPSMAs-7 to -10 showed improved PSMA binding affinities (IC_50_s) and internalization rates, which were, compared with the reference ligands ^18^F-DCFPyL and ^18^F-PSMA-1007, similar or even better. The superiority of EuE-based inhibitors derivatized in the same manner as described for rhPSMA-7 to -10 has been previously reported in a detailed study on the structure–activity relationship of EuE- and KuE-based PSMA inhibitors conducted by Babich et al. (37). In a series of otherwise identical PSMA inhibitors, Glu(Lys(R))-urea-Glu–based inhibitors, comprising a free carboxylate (of Lys) in close proximity to the inhibitor motif, showed the highest affinities. The authors speculated that the EuE motif and the free carboxylate of Lys may increase the ligand interaction with PSMA (37). Regarding unspecific uptake in nontarget tissues and organs, all rhPSMA and both fluorinated reference ligands showed similar uptake profiles in most tissues. Most probably as a result of their high HSA binding and low lipophilicity, the blood levels at 1 h after injection were generally slightly higher for rhPSMA ligands, whereas the liver uptake was lower compared with the reference ligands. Not unexpectedly, the most lipophilic tracer in this study, ^18^F-PSMA-1007 (log P = −1.6), showed the highest uptake in almost all organs and tissues.

Because the internalizations of uncomplexed ^18^F-labeled ligands were superior to the respective Ga-chelated counterpart (Fig. 3B), the biodistribution profiles of ^68^Ga-^19^F-rhPSMA-7 and ^18^F-rhPSMA-7 with free DOTAGA chelator were also compared. Although the in vitro parameters seem to favor ^18^F-rhPSMA-7, the tumor uptake of the uncomplexed ligand was unexpectedly low, despite its 1.3-fold-higher internalization and comparable PSMA affinity. The exact reason for this finding remains unclear and needs further investigation. The additional free carboxylic acids of the uncomplexed ^18^F-rhPSMA-7 inhibitor also accounted for a 2-fold increased kidney uptake, again demonstrating the negative influence of charges on tracer uptake in the kidneys (38–40).

After completion of this study, an automated production of ^18^F-^nat^Ga-rhPSMA-7 has been successfully developed and established at the Department of Nuclear Medicine, Technical University of Munich and Department of Nuclear Medicine, Ludwig Maximilians University Munich. The results and experience gained from almost 400 routine productions will be described elsewhere. Results on the first clinical evaluation of ^18^F-^nat^Ga-rhPSMA-7 are described in this issue of *The Journal of Nuclear Medicine* by Oh et al. (41), Eiber et al. (42), and Kroenke et al. (43).

In summary, we could demonstrate that rhPSMA inhibitors, as a first series of radiopharmaceuticals developed under the radiohybrid concept, are powerful new inhibitors with equal or even better targeting characteristics than the established ^18^F-PSMA tracers DCFPyL and PSMA-1007. Moreover, such radiohybrids offer the possibility to produce the identical ^68^Ga-labeled ^19^F-^68^Ga-rhPSMA tracers at sites that favor ^68^Ga labeling and the possibility to extend this concept to theranostic radiohybrid radiopharmaceuticals, such as F-Lu-rhPSMA.

## CONCLUSION

The development of an automated production of F-Ga-rhPSMA-7 and F-Ga-rhPSMA-10 is highly warranted and a prerequisite to assess the clinical value of the first ^18^F-rhPSMAs in proof-of-concept studies in men.

## DISCLOSURE

Hans-Jürgen Wester, Alexander Wurzer, and Matthias Eiber have a patent application for rhPSMA. Matthias Eiber and Hans-Jürgen Wester receive funding from the SFB 824 (DFG Sonderforschungsbereich 824, Project B11); Hans-Jürgen Wester receives funding from the SFB 824, Project Z) from the Deutsche Forschungsgemeinschaft, Bonn, Germany. Matthias Eiber received funding from Blue Earth Diagnostics Ltd (licensee for rhPSMA) as part of an academic collaboration and is consultant for Blue Earth Diagnostics Ltd. Hans-Jürgen Wester is founder, shareholder and scientific advisor of Scintomics GmbH, Fuerstenfeldbruck, Germany. No other potential conflicts of interest relevant to this article exist.

**KEY POINTS**

**QUESTION:** Is it possible to design SiFA-conjugated PSMA inhibitors with promising characteristics by introduction of a chelate into the same molecule?
**PERTINENT FINDINGS:** The results of this study confirm the working hypothesis. Especially ^18^F-^nat^Ga-rhPSMA-7 meets all major preclinical and pharmaceutical requirements for further assessment in humans.
**IMPLICATIONS FOR PATIENT CARE:** This study on rhPSMAs and the entire radiohybrid concept could open new perspectives in prostate cancer theranostics.
