# Supplementary material for: Radiohybrid Ligands: A Novel Tracer Concept Exemplified by 18F- or 68Ga-Labeled rhPSMA Inhibitors
Source: J Nucl Med. 2020 May;61(5):735–42. doi: 10.2967/jnumed.119.234922 (PMC7198388; doi:10.2967/jnumed.119.234922)
Supplement: Supplementary file 1 [file jnm234922SupplementalData.pdf]

Radiohybrid ligands: a novel tracer concept exemplified by the development, radiolabeling and comparative preclinical evaluation of hybrid ^{18}F- or ^{68}Ga-labeled PSMA-inhibitors

Alexander Wurzer[1], Daniel Di Carlo[1], Alexander Schmidt[1], Roswitha Beck[1], Matthias Eiber[2], Markus Schwaiger[2] and Hans-Jürgen Wester[1]

[1] Chair of Pharmaceutical Radiochemistry, Technical University of Munich, Garching, Germany;

[2] Department of Nuclear Medicine, Klinikum rechts der Isar, Technical University of Munich, Munich, Germany.

Corresponding author:

Hans-Jürgen Wester, PhD
Phone: +49.89.289.12203
Technical University of Munich,
Chair of Pharmaceutical Radiochemistry,
Walther-Meißner-Str. 3
85748 Garching
GERMANY
Fax: +49.89.289.12204
Email: H.J.Wester@tum.de

1. General Information

The Fmoc-(9-fluorenylmethoxycarbonyl-) and all other protected amino acid analogs were purchased from Bachem (Bubendorf, Switzerland) or Iris Biotech (Marktredwitz, Germany). The tritylchloride polystyrene

(TCP) resin was obtained from PepChem (Tübingen, Germany). Chematech (Dijon, France) delivered the chelators DOTAGA (2-(4,7,10-tris(carboxymethyl)-1,4,7,10-tetraazacyclododecan-1-yl)pentanedioic acid), DOTA (1,4,7,10-Tetraazacyclododecane-1,4,7,10-tetraacetic acid), NOTA (2,2′,2”-(1,4,7-triazacyclononane-1,4,7-triyl)triacetic acid) and derivatives thereof. All necessary solvents and other organic reagents were purchased from either, Alfa Aesar (Karlsruhe, Germany), Sigma-Aldrich (Munich, Germany), Fluorochem (Hadfield, UK) or VWR (Darmstadt, Germany).

The *t*Bu-protected PSMA-addressing binding motifs, Lys-urea-Glu ((*t*BuO)KuE(O*t*Bu)$_2$) and Glu-urea-Glu ((tBuO)EuE(OtBu)$_2$) as well as the derivative PfpO-Sub-(*t*BuO)KuE(O*t*Bu)$_2$ (Pentafluorophenyl-suberic acid active ester of the *t*Bu-protected EuK binding motif) were prepared in analogy to previously described procedures (*1*-*3*). Synthesis of the Silicon-Fluoride-Acceptor, 4-(di-tert-butylfluorosilyl)benzoic acid (SiFA-BA) and the alkyne-functionalized TRAP chelator (1,4,7-triazacyclononane-1,4,7-tris[methyl(2-carboxyethyl)phosphinic acid) were performed according to the literature protocols (*4*,*5*).

Solid phase synthesis of the peptides was carried out by manual operation using an syringe shaker (Intelli, Neolab, Heidelberg, Germany). Analytical and preparative reversed-phase high-pressure liquid chromatography (RP-HPLC) were performed using Shimadzu gradient systems (Shimadzu, Neufahrn, Germany), each equipped with a SPD-20A UV/Vis detector (220 nm, 254 nm). A Nucleosil 100 C18 (125 × 4.6 mm, 5 µm particle size) column (CS Chromatographie Service, Langerwehe, Germany) was used for analytical measurements at a flow rate of 1 mL/min. Both specific gradients and the corresponding retention times t_R are cited in the text. Preparative HPLC purification was done with a Multospher 100 RP 18 (250 × 10 mm, 5 µm particle size) column (CS Chromatographie Service, Langerwehe, Germany) at a constant flow rate of 5 mL/min. Analytical and preparative radio RP-HPLC was performed using a Nucleosil 100 C18 (5 µm, 125 × 4.0 mm) column (CS Chromatographie Service, Langerwehe, Germany). Eluents for all HPLC operations were water (solvent A) and acetonitrile (solvent B), both containing 0.1% trifluoroacetic acid. Radioactivity was detected through connection of the outlet of the UV-photometer to a HERM LB 500 NaI detector (Berthold Technologies, Bad Wildbad, Germany). Electrospray ionization-mass spectra for characterization of the substances were acquired on an expression^L CMS mass spectrometer

(Advion, Harlow, United Kingdom). NMR spectra were recorded on Bruker (Billerica, USA) AVHD-300 or AVHD-400 spectrometers at 300 K. Activity quantification was performed using a 2480 WIZARD[2] automatic gamma counter (PerkinElmer, Waltham, United States). Radio-thin layer chromatography (TLC) was carried out with a Scan-RAM detector (LabLogic Systems, Sheffield, United Kingdom).

2. General Procedures (GP) for peptide synthesis

TCP-resin loading (GP1)

Loading of the tritylchloride polystyrene (TCP) resin with a Fmoc-protected amino acid (AA) was carried out by stirring a solution of the TCP-resin (1.60 mmol/g) and Fmoc-AA-OH (1.5 eq.) in anhydrous DCM with DIPEA (3.8 eq.) at room temperature for 2 h. Remaining tritylchloride was capped by the addition of methanol (2 mL/g resin) for 15 min. Subsequently the resin was filtered and washed with DCM (2 × 5 mL/g resin), DMF (2 × 5 mL/g resin), methanol (5 mL/g resin) and dried *in vacuo*. Final loading *l* of Fmoc-AA-OH was determined by the following equation:

$$l\left[\frac{mmol}{g}\right]=\frac{(m_2-m_1)\times 1000}{(M_W-M_{HCl})\,m_2}$$

m_2 = mass of loaded resin [g]
m_1 = mass of unloaded resin [g]
M_W = molecular weight of AA [g/mol]
M_{HCl} = molecular weight of HCl [g/mol]

On-resin Amide Bond Formation (GP2)

For conjugation of a building block to the resin-bound peptide, a mixture of TBTU with HOBt or HOAt is used for pre-activation of the carboxylic with DIPEA as a base in DMF (10 mL/g resin). After 5 min at rt, the solution is added to the swollen resin. The exact stoichiometry and reaction time for each conjugation step is given in the respective synthesis protocols. After reaction, the resin was washed with DMF (6 × 5 mL/g resin).

On-resin Fmoc-deprotection (GP3)

The resin-bound Fmoc-peptide was treated with 20% piperidine in DMF (*v/v*, 8 mL/g resin) for 5 min and subsequently for 15 min. Afterwards, the resin was washed thoroughly with DMF (8 × 5 mL/g resin).

On-resin Dde-deprotection (GP4)

The Dde-protected peptide (1.0 eq.) was dissolved in a solution of 2% hydrazine monohydrate in DMF (*v/v*, 5 mL/g resin) and shaken for 20 min (GP4a). In the case of present Fmoc-groups, Dde-deprotection was performed by adding a solution of imidazole (0.92 g/g resin), hydroxylamine hydrochloride (1.26 g/g reisn) in NMP (5.0 mL/g resin) and DMF (1.0 mL/g resin) for 3 h at room temperature (GP4b). After deprotection the resin was washed with DMF (8 × 5 mL/g resin).

On-resin Allyl-deprotection (GP5)

The allyl-protecting group was removed by the addition of triisopropylsilane (TIPS) (50.0 eq.) and tetrakis(triphenylphosphine)palladium(0) ($Pd(PPh_3)_4$) (0.3 eq.) dissolved in DCM (8 mL/g resin). After 1.5 h at room temperature, the resin was washed with DCM (6 × 5 mL/g resin) and DMF (6 × 5 mL/g resin).

tBu/Boc deprotection (GP6)

Removal of *t*Bu/Boc-protecting groups was carried out by dissolving the crude product in a mixture of TFA/TIPS/water (*v/v/v*; 95/2.5/2.5) and stirring for 1-6 h at rt. Product formation was monitored by HPLC-analysis. After removing TFA under a stream of nitrogen, the residue was dissolved in a mixture of *tert*-butanol and water. After lyophilisation the crude peptide was obtained.

Peptide cleavage from the resin (GP7)

a. Preservation of acid labile protecting groups (GP7a): The resin-bound peptide was dissolved in a mixture of DCM/TFE/AcOH (*v/v/v*; 6/3/1, 8 mL/g resin) and shaken for 30 min. The solution containing the fully protected peptide was filtered off and the resin was treated with another portion of the cleavage solution for 30 min. Both fractions were combined and acetic acid was removed under reduced pressure by successively adding toluene and water. After lyophilisation of remaining water, the crude fully protected peptide was obtained.

b. Deprotection of all acid labile protecting groups (GP7b): The fully protected resin-bound peptide was dissolved in a mixture of TFA/TIPS/water (*v/v/v*; 95/2.5/2.5) and shaken for 30 min. The solution was filtered off and the resin was treated in the same way for another 30 min. Both filtrates were combined, stirred for additional 1-6 h at rt. Product formation was monitored by HPLC. After removing TFA under a stream of nitrogen, the residue was dissolved in a mixture of *tert*-butanol and water and freeze-dried.

Conjugation of PfpO-Sub-(tBuO)KuE(OtBu)$_2$ to the peptide (GP8)

The *N*-terminal deprotected peptide (1.0 eq.) was added to a solution of **3** (1.2 eq.) in DMF (approx. 0.1 mL/ mg peptide) and TEA (8 eq.) was added. After stirring the solution for 2 h at rt, DMF was removed *in vacuo*. For cleavage of the *t*Bu-esters, TFA was added and the solution was stirred for 45 min at rt. After removing TFA under a stream of nitrogen, the crude product was purified by RP-HPLC.

Coupling of propargyl-TRAP to the peptide (GP9)

For conjugation of azide-functionalized peptides to propargyl-TRAP via copper(I)-catalyzed alkyne-azide cycloaddition a previously developed procedure was applied (*5*). Briefly, propargyl-TRAP (1.0 eq.) was dissolved in water (40 mM solution) and combined with a solution of the peptide (1.1 eq.) in a 1:1 (*v/v*) mixture of *t*BuOH and water (approx. 20-40 mM). Subsequently, a solution of sodium ascorbate (0.5 M, 50 eq.) in water was added. In order to start the reaction, an aqueous solution of $Cu(OAc)_2 \cdot H_2O$ (0.05 M,

1.2 eq.) was added, which resulted in a brown precipitate that dissolved after stirring in a clear green solution. For demetallation of TRAP, an aqueous solution of NOTA (8 mM, 12 eq.) was added and the pH was adjusted to 2.2 with 1 M aq. HCl. After either 1 h at 60 °C, or 48 h at rt the mixture was directly subjected to preparative HPLC purification.

Conjugation of DOTAGA anhydride (GP10)

The *N*-terminal deprotected peptide (1.0 eq.) was dissolved together with DOTAGA-anhydride (1.5 eq.) and DIPEA (10.0 eq.) in dry DMF. After stirring the reaction mixture overnight at rt, DMF was removed *in vacuo*, yielding the crude product.

3. Synthesis of rhPSMA ligands

rhPSMA-5

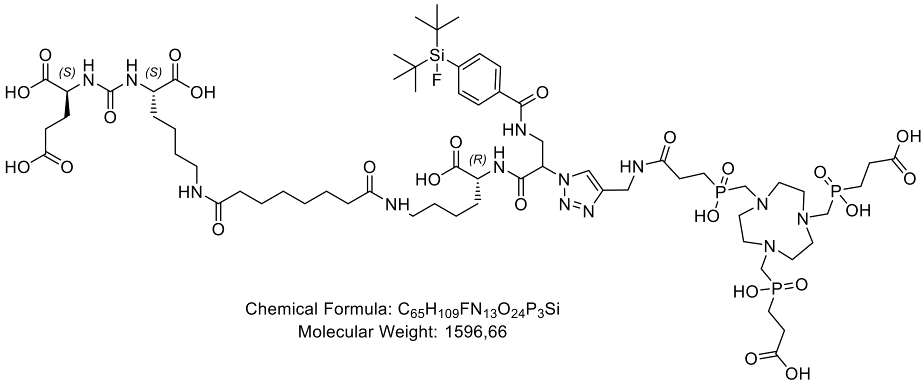

Supplemental Figure 1. Structural formula of uncomplexed rhPSMA-5.

Synthesis of rhPSMA-5 was carried out by applying the general methods and procedures mentioned before. Shortly, resin-bound Fmoc-D-Lys(Boc)-OH was Fmoc-deprotected with 20% piperidine in DMF (GP3) and conjugated to N_3-L-Dap(Fmoc)-OH (2.0 eq.) with HATU (3.0 eq.), HOAt (3.0 eq.) and DIPEA (6.0 eq.) for 2 h in DMF (GP2). After cleavage of the Fmoc-group (GP3), SiFA-BA (1.5 eq.) was added with HOBt (1.5 eq.), TBTU (1.5 eq.) and DIPEA (4.5 eq.) in DMF for 2 h (GP2). Subsequent cleavage from the resin

with TFA yielded the fully deprotected peptide backbone (GP7b). For conjugation of the EuK-moiety, PfpO-Sub-(*t*BuO)KuE(O*t*Bu)$_2$ (1.2 eq.) was added in a mixture of TEA (8 eq.) and DMF (GP8). Cleavage of the *t*Bu-esters was performed by adding TFA (GP6). In a final step the purified peptide (1.1 eq.) was reacted with propargyl-TRAP (1.0 eq.) in a *copper(I)-catalyzed* click reaction, as mentioned above (GP9). After RP-HPLC purification rhPSMA-5 (4%) was obtained as a colourless solid. HPLC (10 to 90% B in 15 min): t_R = 8.5 min. Calculated monoisotopic mass ($C_{65}H_{109}FN_{13}O_{24}P_3Si$): 1595.7; found: m/z = 1596.5 $[M+H]^+$, 799.1 $[M+2H]^{2+}$.

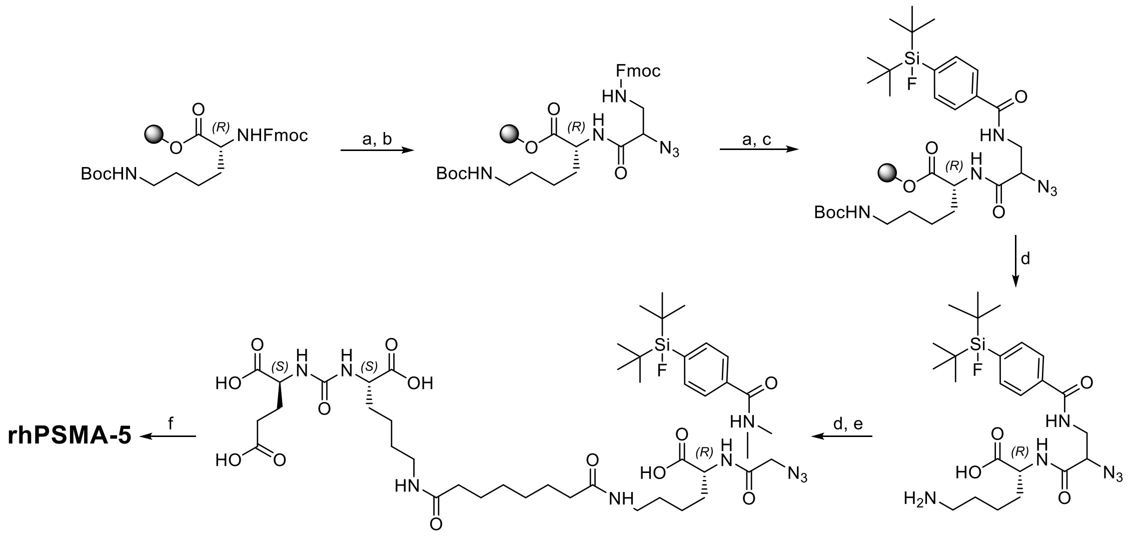

Supplemental Figure 2. Synthesis of rhPSMA-5: a) 20% piperidine (DMF); b) N_3-L-Dap(Fmoc)-OH, HATU, HOAt, DIPEA (DMF); c) SIFA-BA, HOBt, TBTU, DIPEA (DMF); d) TFA; e) PfpO-Sub-(*t*BuO)-KuE(O*t*Bu)$_2$, TEA (DMF)*;* f) propargyl-TRAP, $Cu(OAc)_2 \cdot H_2O$, sodium ascorbate (*t*BuOH, H_2O).

rhPSMA-6

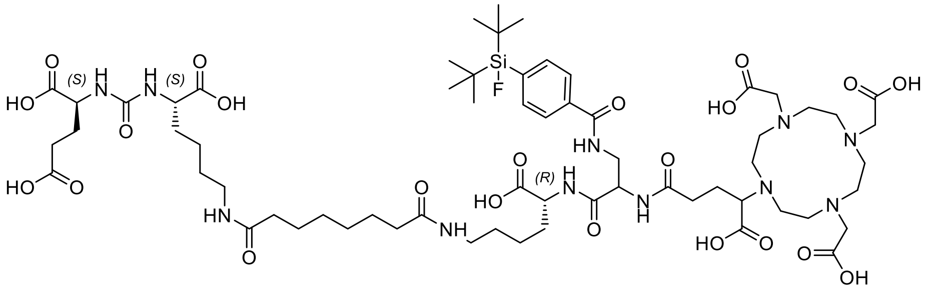

Chemical Formula: $C_{63}H_{102}FN_{11}O_{22}Si$
Molecular Weight: 1412,65

Supplemental Figure 3. Structural formula of uncomplexed rhPSMA-6.

Resin-bound Fmoc-D-Lys(Boc) was Fmoc deprotected with 20% piperidine in DMF (GP3) and Fmoc-D-Dap(Dde)-OH (2.0 eq.) was conjugated applying HOBt (2.0 eq.), TBTU (2.0 eq.) and DIPEA (6.0 eq.) in DMF for 2 h (GP2). After orthogonal Dde-deprotection with imidazole and hydroxylamine hydrochloride in a mixture of NMP and DMF (GP4b), SIFA-BA (1.5 eq.) was conjugated with HOBt (1.5 eq.), TBTU (1.5 eq.) and DIPEA (4.5 eq.) in DMF for 2 h (GP2). Subsequent Fmoc-deprotection (GP3) and mild cleavage from the resin with TFE and AcOH in DCM (GP7a) yielded the *t*Bu-protected peptide backbone. Condensation of DOTAGA-anhydride (1.5 eq.) was performed by adding DIPEA (10 eq.) in DMF (GP10). After *t*Bu-deprotection in TFA (GP6), the PfpO-Sub-(tBuO)KuE$(OtBu)_2$ moiety (1.2 eq.) was added in a mixture of TEA (8 eq.) and DMF (GP8). Final cleavage of the *t*Bu-esters in TFA (GP6) and RP-HPLC purification yielded rhPSMA-6 (70%) as a colorless solid. HPLC (10 to 90% B in 15 min): t_R = 9.1 min. Calculated monoisotopic mass ($C_{63}H_{102}FN_{11}O_{22}Si$): 1411.7; found: m/z = 1412.3 $[M+H]^+$, 706.8 $[M+2H]^{2+}$.

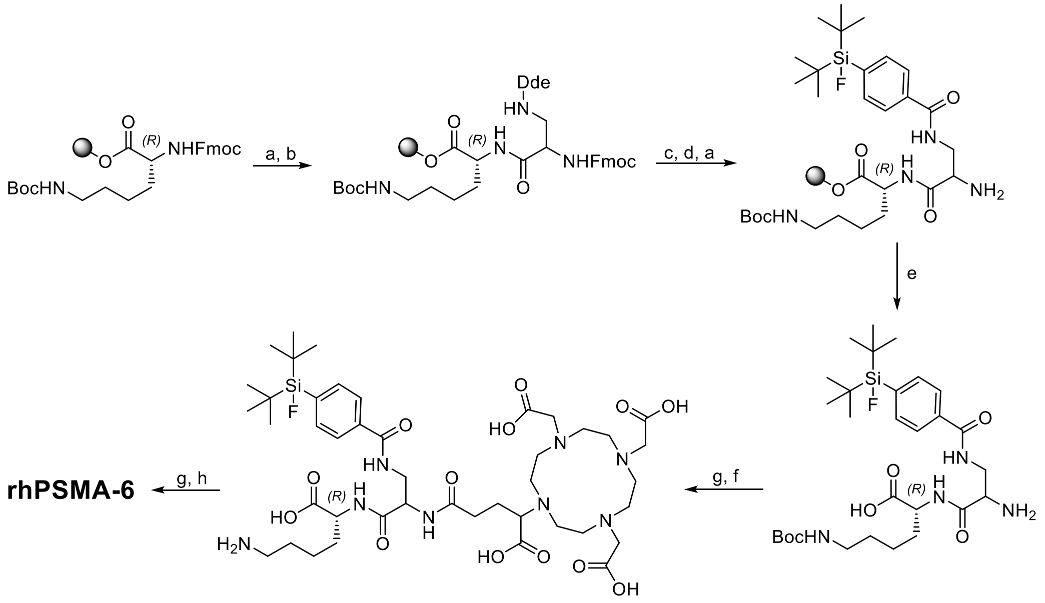

Supplemental Figure 4. Synthesis of rhPSMA-6: a) 20% piperidine (DMF); b) Fmoc-D-Dap(Dde)-OH, HOBt, TBTU, DIPEA (DMF); c) imidazole, hydroxylamine hydrochloride (NMP, DMF); d) SIFA-BA, HOBt, TBTU, DIPEA (DMF); e) TFE, AcOH (DCM); f) DOTAGA-anhydride, DIPEA (DMF); g) TFA; h) PfpO-Sub-(*t*BuO)KuE(O*t*Bu)$_2$, TEA (DMF).

rhPSMA-7

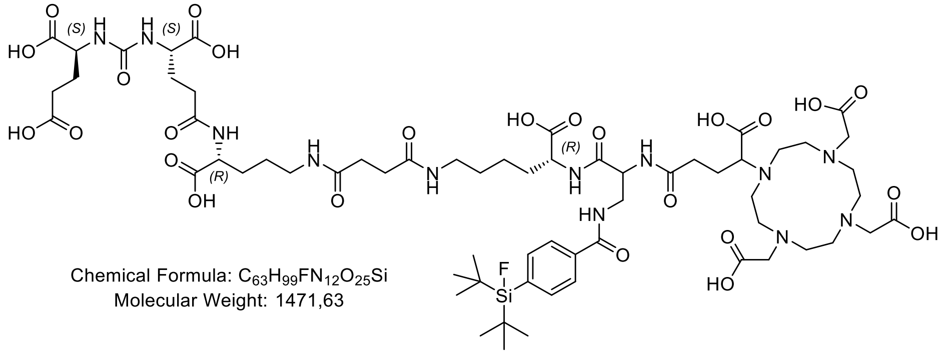

Supplemental Figure 5. Structural formula of uncomplexed rhPSMA-7.

Resin-bound Fmoc-D-Orn(Dde)-OH was Fmoc-deprotected with 20% piperidine in DMF (GP3) and (*t*BuO)EuE(O*t*Bu)$_2$ (2.0 eq.) was conjugated with HOBt (2.0 eq.), TBTU (2.0 eq.) and DIPEA (6.0 eq.) in DMF for 4.5 h (GP3). After cleavage of the Dde-group with a mixture of 2% hydrazine in DMF (GP4a), a solution of succinic anhydride (7.0 eq.) and DIPEA (7.0 eq.) in DMF was added and reacted for 2.5 h. Conjugation of Fmoc-D-Lys-OAll·HCl (1.5 eq.) was achieved by adding a mixture of HOBt (1.5 eq.), TBTU (1.5 eq.) and DIPEA (4.5 eq.) in DMF for 2 h (GP2). After cleavage of the Fmoc-group with 20% piperidine in DMF (GP3), the free amine was conjugated to Fmoc-D-Dap(Dde)-OH (2.0 eq.) after pre-activation of the amino acid in a mixture of HOBt (2.0 eq.), TBTU (2.0 eq.) and DIPEA (6.0 eq.) in DMF for 2 h (GP2). Following orthogonal Dde-deprotection was done using imidazole and hydroxylamine hydrochloride dissolved in a mixture of NMP and DMF (GP4b). SiFA-BA (1.5 eq.) was reacted with the free amine of the side chain with HOBt (1.5 eq.), TBTU (1.5 eq.) and DIPEA (4.5 eq.) as activation reagents in DMF for 2 h (GP2). The allyl-protecting group was removed by the addition of TIPS (50.0 eq.) and $Pd(PPh_3)_4$ (0.3 eq.) dissolved in DCM (GP5). After Fmoc-deprotection with piperidine (GP3), the peptide was cleaved from the resin under preservation of the acid labile protecting groups by using a mixture of TFE and AcOH in DCM (GP7a). Final condensation of DOTAGA-anhydride (1.5 eq.) was achieved with piperidine (10 eq.) in DMF (GP10). After cleavage of the *t*Bu-esters of the EuE-moiety with TFA (GP6), the crude peptide was purified by RP-HPLC, yielding rhPSMA-7 (24%) as a colorless solid. HPLC (10 to 70% B in 15 min): t_R = 10.4 min. Calculated monoisotopic mass ($C_{63}H_{99}FN_{12}O_{25}Si$): 1470.7; found: m/z = 1471.8 $[M+H]^+$, 736.7 $[M+2H]^{2+}$.

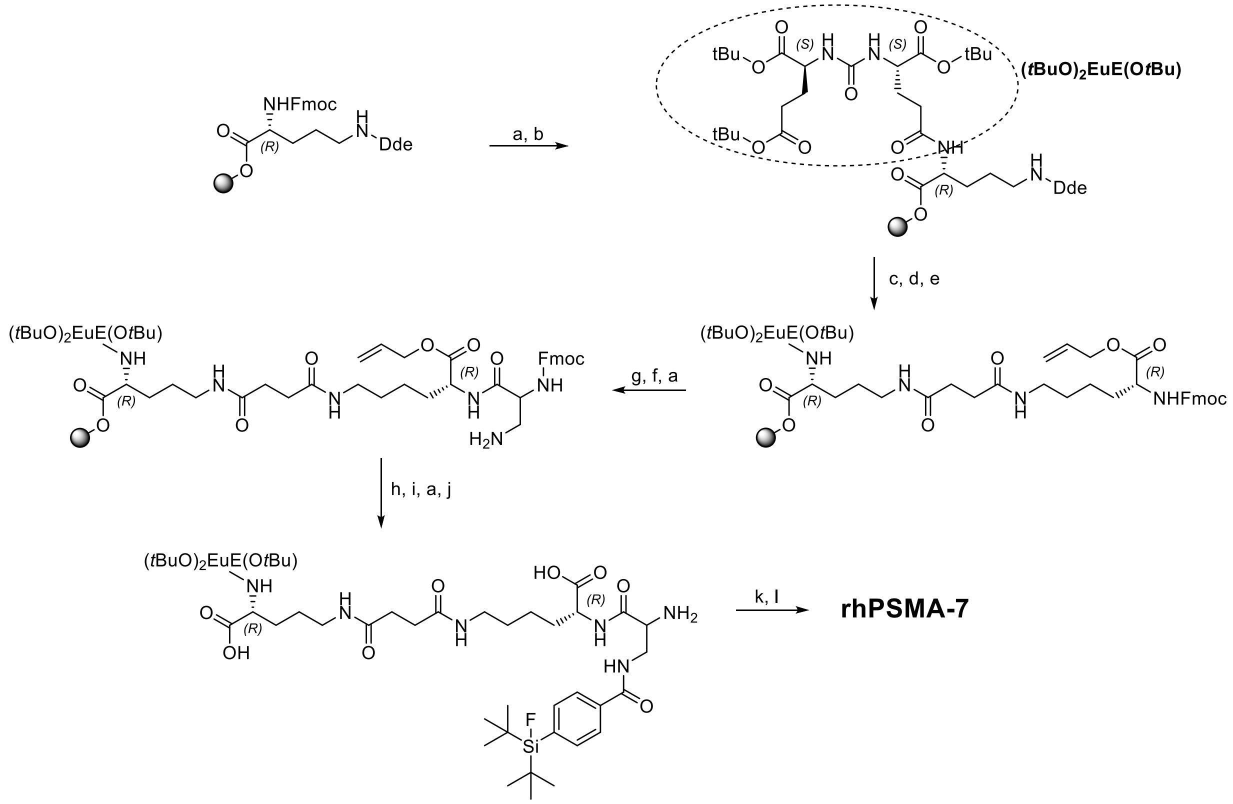

Supplemental Figure 6. Synthesis of rhPSMA-7: a) 20% piperidine (DMF); b) (tBuO)EuE(OtBu)$_2$, HOBt, TBTU, DIPEA (DMF); c) 2% hydrazine (DMF); d) succinic anhydride, DIPEA (DMF); e) Fmoc-D-Lys-OAll·HCl, HOBt, TBTU, DIPEA (DMF); f) Fmoc-D-Dap(Dde)-OH, HOBt, TBTU, DIPEA (DMF); g) imidazole, hydroxylamine hydrochloride (NMP, DMF); h) SiFA-BA, HOBt, TBTU, DIPEA (DMF); i) TIPS, Pd(PPh$_3$)$_4$ (DCM); j) TFE, AcOH (DCM); k) DOTAGA-anhydride, DIPEA (DMF); l) TFA.

rhPSMA-8

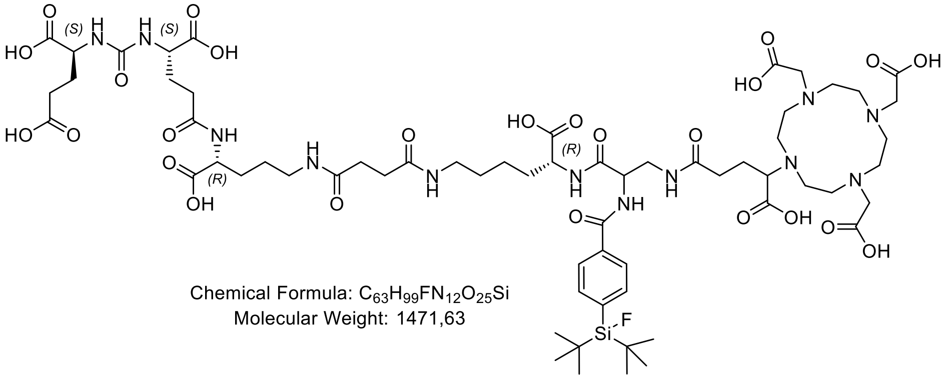

Supplemental Figure 7. Structural formula of uncomplexed rhPSMA-8.

Synthesis of rhPSMA-8, was carried out as described for rhPSMA-7, with one deviation; After conjugation of Fmoc-D-Dap(Dde)-OH, the Fmoc-protecting group was cleaved with piperidine in DMF (GP3) and SiFA-BA was reacted with the free N-terminus of the peptide (GP2). After removing the allyl-protecting group with TIPS (50.0 eq.) and $Pd(PPh_3)_4$ (0.3 eq.) dissolved in DCM (GP5), the remaining Dde-group was cleaved by a solution of imidazole and hydroxylamine hydrochloride dissolved in NMP and DMF (GP4b). Following conjugation of DOTAGA and final deprotection were carried out as described for rhPSMA-7. After RP-HPLC purification, rhPSMA-8 (11%) was obtained as a colorless solid. HPLC (10 to 70% B in 15 min): t_R = 10.4 min. Calculated monoisotopic mass ($C_{63}H_{99}FN_{12}O_{25}Si$): 1470.7; found: m/z = 1471.7 $[M+H]^+$, 736.8 $[M+2H]^{2+}$.

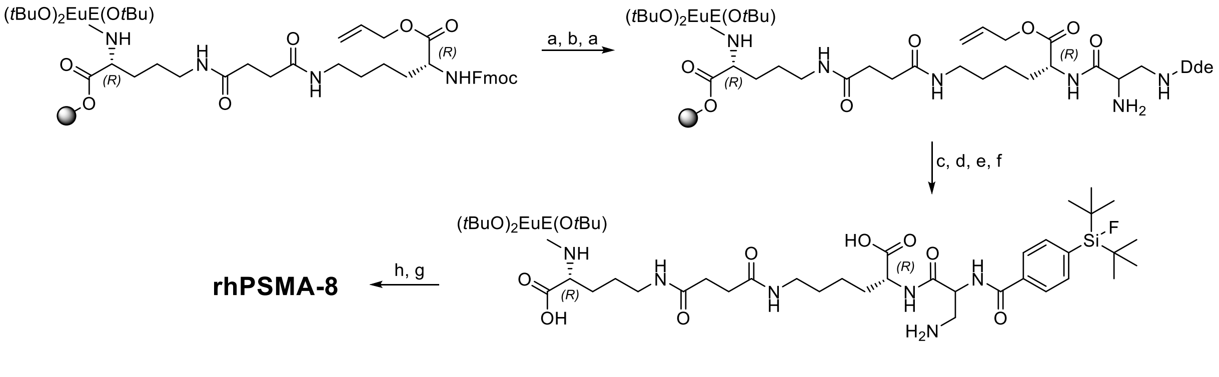

Supplemental Figure 8. Synthesis of rhPSMA-8: a) 20% piperidine (DMF); b) Fmoc-D-Dap(Dde)-OH, HOBt, TBTU, DIPEA (DMF); c) SIFA-BA, HOBt, TBTU, DIPEA (DMF); d) TIPS, $Pd(PPh_3)_4$ (DCM); e) imidazole, hydroxylamine hydrochloride (NMP, DMF); f) TFE, AcOH (DCM); g) DOTAGA-anhydride, DIPEA (DMF); h) TFA.

rhPSMA-9

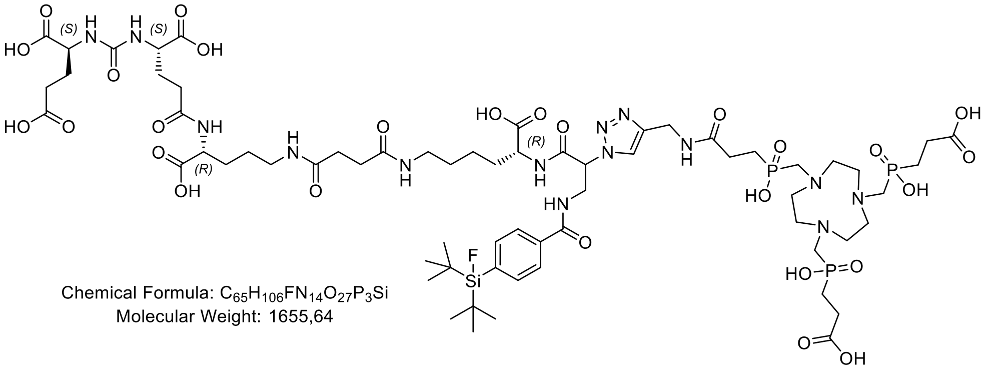

Supplemental Figure 9. Structural formula of uncomplexed rhPSMA-9.

The peptide backbone of rhPSMA-9 was prepared analogously to rhPSMA-7 and 8. A difference was the use of N_3-L-Dap(Fmoc)-OH instead of Fmoc-D-Dap(Dde)-OH, which was required for the final click reaction with propargyl-TRAP. The azido-substituted amino acid (2.0 eq.) was conjugated with HATU (3.0 eq.), HOAt (3.0 eq.) and DIPEA (6.0 eq.) in DMF for 2 h (GP2). After Fmoc-deprotection with 20% piperidine (GP3), SIFA-BA (1.5 eq.) was reacted with HOBt (1.5 eq.), TBTU (1.5 eq.) and DIPEA (4.5 eq.) as activation reagents in DMF for 2 h (GP2). Removal of the allyl-protecting group was performed by the addition of TIPS (50.0 eq.) and $Pd(PPh_3)_4$ (0.3 eq.) dissolved in DCM (GP5). After cleavage from the resin with TFA under concurrent deprotection of all acid labile protecting groups (GP7b), the purified EuE-azido-conjugate (1.1 eq.) was reacted with propargyl-TRAP (1.0 eq.) in a copper(I)-catalyzed click reaction (GP9). RP-HPLC purification yielded rhPSMA-9 (9%) as a colourless solid. HPLC (10 to 90% B in 15 min): t_R = 8.7 min. Calculated monoisotopic mass ($C_{65}H_{106}FN_{14}O_{27}P_3Si$): 1654.6; found: m/z = 1655.6 $[M+H]^+$, 828.4 $[M+2H]^{2+}$.

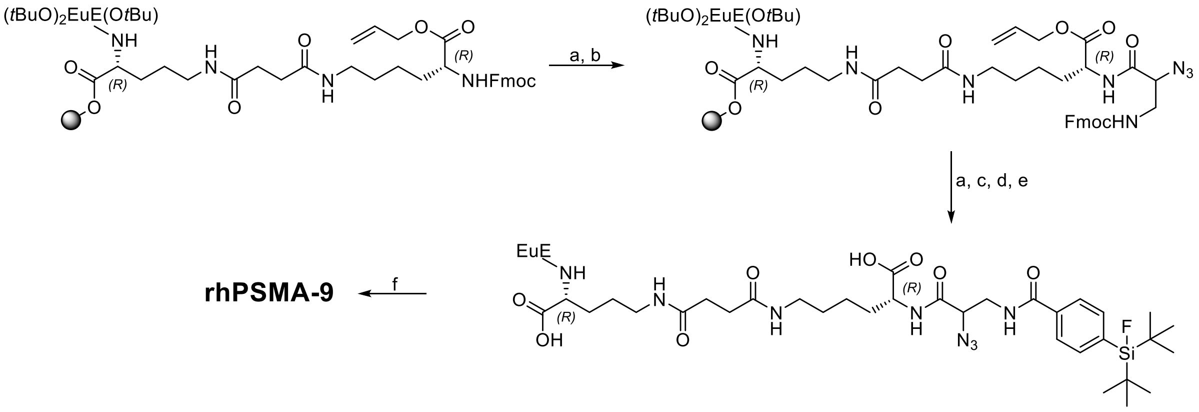

Supplemental Figure 10. Synthesis of rhPSMA-9: a) 20% piperidine (DMF); b) N_3-L-Dap(Fmoc)-OH, HOBt, TBTU, DIPEA (DMF); c) SIFA-BA, HOBt, TBTU, DIPEA (DMF); d) TIPS, $Pd(PPh_3)_4$ (DCM); e) TFA; f) propargyl-TRAP, $Cu(OAc)_2{\cdot}H_2O$, sodium ascorbate (*t*BuOH, H_2O).

rhPSMA-10

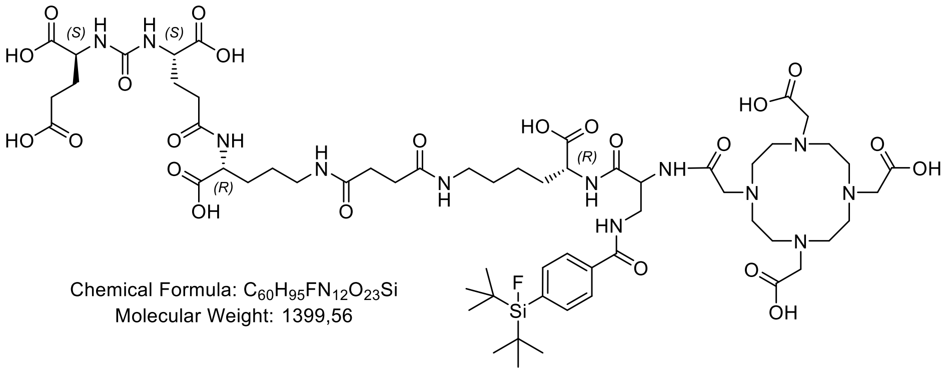

Supplemental Figure 11. Structural formula of uncomplexed rhPSMA-10.

rhPSMA-10 was synthesized in analogy to rhPSMA-7, by using DOTA instead of DOTAGA, starting from resin-bound ((*t*Bu_2)EuE(*t*Bu)-orn-succinic acid. Conjugation of Fmoc-D-Lys(O*t*Bu)·HCl (1.5 eq.) was achieved by adding a mixture of HOBt (1.5 eq.), TBTU (1.5 eq.) and DIPEA (4.5 eq.) in DMF for 2 h (GP2). After cleavage of the Fmoc-group (GP3), Fmoc-D-Dap(Dde)-OH (2.0 eq.) was pre-activated in a mixture of HOAt (2.0 eq.), TBTU (2.0 eq.) and DIPEA (6.0 eq.) in DMF and added to the resin-bound peptide for 2.5 h (GP2). Following orthogonal Dde-deprotection was done using imidazole and hydroxylamine hydrochloride dissolved in a mixture of NMP and DMF for 3 h (GP4b). SiFA-BA (1.5 eq.) was reacted with

the free amine of the side chain with HOAt (1.5 eq.), TBTU (1.5 eq.) and DIPEA (4.5 eq.), as activation reagents in DMF for 2 h (GP2). After Fmoc-deprotection with piperidine (GP3), the *tert*-butyl protected chelator, DOTA(*t*Bu)$_3$ was conjugated with HOAt (2.0 eq.), TBTU (2.0 eq.) and DIPEA (6.0 eq.) in DMF for 2.5 h (GP2). Cleavage from the resin with simultaneous deprotection of acid labile protecting groups was performed in TFA for 6 h (GP7b). After HPLC-based purification, rhPSMA-10 (11%) was obtained as a colorless solid. HPLC (10 to 70% B in 15 min): t_R = 10.5 min. Calculated monoisotopic mass ($C_{60}H_{95}FN_{12}O_{23}Si$): 1398.6; found: m/z = 1399.6 $[M+H]^+$, 700.6 $[M+2H]^{2+}$.

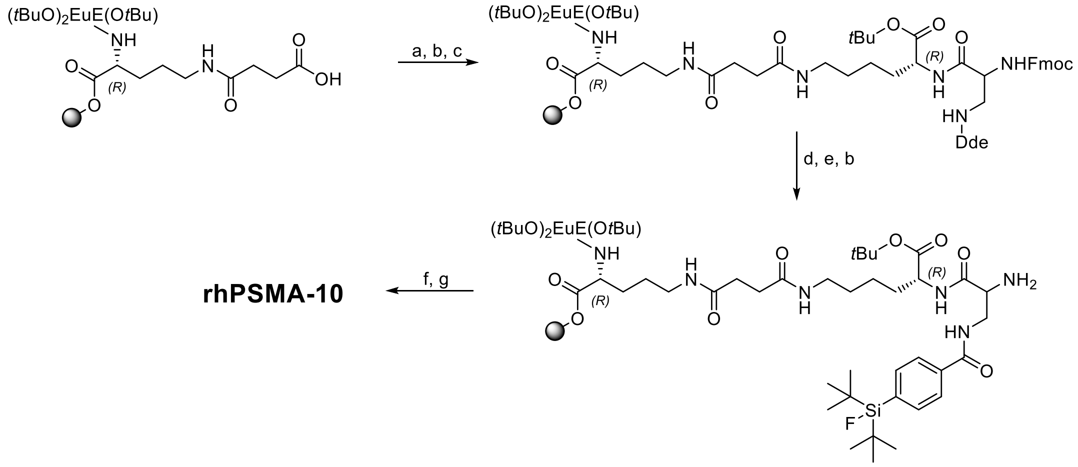

Supplemental Figure 12. Synthesis of rhPSMA-10: a) Fmoc-D-Lys(O*t*Bu)·HCl, HOBt, TBTU, DIPEA (DMF) b) 20% piperidine (DMF); c) Fmoc-D-Dap(Dde)-OH, HOBt, TBTU, DIPEA (DMF); d) imidazole, hydroxylamine hydrochloride (NMP, DMF); e) SiFA-BA, HOBt, TBTU, DIPEA (DMF); f) DOTA(*t*Bu)$_3$, HOBt, TBTU, DIPEA (DMF); g) TFA.

4. Synthesis of cold Gallium complexes

Synthesis of ^{nat}Ga-TRAP complexes

500 µL of a 2 mM stock solution of the rhPSMA precursor (1.0 eq.) in DMSO was combined with 75 µL of a 20 mM $Ga(NO_3)_3$ (1.5 eq.) solution in water. Complexation occurred instantaneously at room temperature. If required, purification of the crude ligand was performed by RP-HPLC.

^{nat}**Ga-rhPSMA-5:** *HPLC* (10 to 90% B in 15 min): t_R = 9.5 min. Calculated monoisotopic mass ($C_{65}H_{106}FGaN_{13}O_{24}P_3Si$): 1661.6; found: m/z = 1663.9 $[M+H]^+$, 832.6 $[M+2H]^{2+}$.

^{nat}**Ga-rhPSMA-9:** *HPLC* (10 to 90% B in 15 min): t_R = 9.0 min. Calculated monoisotopic mass ($C_{65}H_{103}FGaN_{14}O_{27}P_3Si$): 1720.5; found: m/z = 1720.8 $[M+H]^+$, 861.1 $[M+2H]^{2+}$.

Synthesis of ^{nat}*Ga-DOTAGA and* ^{nat}*Ga-DOTA complexes*

500 µL of a 2 mM stock solution of the rhPSMA precursor (1.0 eq.) in DMSO was combined with 150 µL of a 20 mM $Ga(NO_3)_3$ solution (3.0 eq.) in water. The reaction mixture was heated for 30 min at 75 °C. If required, the complexed compound was purified by RP-HPLC.

^{nat}**Ga-rhPSMA-6:** HPLC (10 to 90% B in 15 min): t_R = 9.1 min. Calculated monoisotopic mass ($C_{63}H_{99}FGaN_{11}O_{22}Si$): 1477.6; found: m/z = 1479.5 $[M+H]^+$, 740.2 $[M+2H]^{2+}$.

^{nat}**Ga-rhPSMA-7:** HPLC (10 to 70% B in 15 min): t_R = 10.4 min. Calculated monoisotopic mass ($C_{63}H_{96}FGaN_{12}O_{25}Si$): 1536.6; found: m/z = 1539.4 $[M+H]^+$, 770.3 $[M+2H]^{2+}$.

^{nat}**Ga-rhPSMA-8:** HPLC (10 to 70% B in 15 min): t_R = 10.4 min. Calculated monoisotopic mass ($C_{63}H_{96}FGaN_{12}O_{25}Si$): 1536.6; found: m/z = 1539.1 $[M+H]^+$, 770.5 $[M+2H]^{2+}$.

^{nat}**Ga-rhPSMA-10:** HPLC (10 to 70% B in 15 min): 9.5 min. Calculated monoisotopic mass ($C_{60}H_{92}FGaN_{12}O_{23}Si$): 1464.5; found: m/z = 1467.3 $[M+H]^+$, 733.9 $[M+2H]^{2+}$.

5. Radiolabelling

^{68}Ga-labelling was performed using an automated system (GallElut$^+$ by Scintomics, Germany) as described previously (*6*). Briefly, the ^{68}Ge/^{68}Ga-generator with SnO_2 matrix (IThemba LABS) was eluted with 1.0 M aqueous HCl, from which a fraction (1.25 mL) of approximately 80% of the activity (500–700 MBq), was transferred into a reaction vial (ALLTECH, 5 mL). The reactor was loaded before elution with 2–5 nmol of respective chelator conjugate in an aqueous 2.7 M HEPES solution (DOTA/DOTAGA-conjugates: 900 µL, TRAP-conjugates: 400 µL). After elution the vial was heated for 5 minutes at 95 °C. Purification was done by passing the reaction mixture over a solid phase extraction cartridge (C 8 light, SepPak), which was purged

with water (10 mL). The purified product was eluted with 50% aqueous ethanol (2 mL), phosphate buffered saline (PBS, 1 mL) and again water (1 mL). After removing ethanol *in vacuo*, purity of the radiolabelled compounds was determined by radio-HPLC and radio-TLC (ITLC-SG chromatography paper, mobile phase: 0.1 M trisodium citrate and Silica gel 60 RP-18 F_{254}s, mobile phase: 3:2 mixture (v/v) of MeCN in H_2O supplemented with 10% of 2 M NaOAc solution and 1% of TFA).

6. Human Serum Albumin (HSA) Binding

HSA binding of the PSMA-addressing ligands was determined according to a previously published procedure via HPLC (*7*). A Chiralpak HSA column (50 x 3 mm, 5 µm, H13H-2433, Daicel, Tokyo, Japan) was used at a constant flow rate of 0.5 mL/min at rt. Mobile phase A was a freshly prepared 50 mM aqueous solution of NH_4OAc (pH 6.9) and mobile phase B was isopropanol (HPLC grade, VWR, Germany). The applied gradient for all experiments was 100% A (0 to 3 min), followed by 80% A (3 to 40 min). Prior to the experiment, the column was calibrated using nine reference substances with a HSA binding, known from literature, in the range of 13 to 99% (*7*,*8*). All substances, including the examined PSMA ligands, were dissolved in a 1:1 mixture (v/v) of isopropanol and a 50 mM aqueous solution of NH_4OAc (pH 6.9) with a final concentration of 0.5 mg/mL. Non-linear regression was established with the OriginPro 2016G software (Northampton, United States).

7. *In vitro* Experiments

Affinity determinations (IC_{50})

The PSMA affinity (IC_{50}) determinations and synthesis of the radioiodinated reference ligand (((S)-1-carboxy-5-(4-^{125}I-iodobenzamido)pentyl)carbamoyl)-*L*-glutamic acid, ((^{125}I-I-BA)KuE) were performed as described previously (*1*). Briefly, the respective ligand was diluted (serial dilution 10^{-4} to 10^{-10}) in Hank's balanced salt solution (HBSS, Biochrom). In the case of metal-complexed ligands, the crude reaction mixture containing the purified metal-complexed inhibitor and remaining metal nitrate, was diluted

analogously without further purification.. Cells were harvested 24 ± 2 hours prior to the experiment and seeded in 24-well plates (1.5 × 10^5 cells in 1 mL/well). After removal of the culture medium, the cells were carefully washed with 500 µL of HBSS, supplemented with 1% bovine serum albumin (BSA, Biowest, Nuaillé, France) and left 15 min on ice for equilibration in 200 µL HBSS (1% BSA). Next, 25 µL per well of solutions, containing either HBSS (1% BSA, control) or the respective ligand in increasing concentration (10^{-10} – 10^{-4} M in HBSS) were added with subsequent addition of 25 µL of ^{125}I-I-BA-KuE (2.0 nM) in HBSS (1% BSA). After incubation on ice for 60 min, the experiment was terminated by removal of the medium and consecutive rinsing with 200 µL of HBSS (1% BSA). The media of both steps were combined in one fraction and represent the amount of free radioligand. Afterwards, the cells were lysed with 250 µL of 1 M aqueous NaOH for at least 10 min. After a washing step (250 µL of 1 M NaOH), both fractions, representing the amount of bound ligand, were united. Quantification of all collected fractions was accomplished in a γ-counter. PSMA-affinity determinations were carried out at least three times per ligand.

Internalization studies

Internalization studies were carried out according to a previously published procedure (*1*). Briefly, LNCaP cells were harvested 24 ± 2 hours before the experiment and seeded in poly-*L*-lysine coated 24-well plates (1.25 × 10^5 cells in 1 mL/well, Greiner Bio-One, Kremsmünster, Austria). After removal of the culture medium, the cells were washed once with 500 µL DMEM-F12 (5% BSA) and left to equilibrate for at least 15 min at 37 °C in 200 µL DMEM-F12 (5% BSA). Each well was treated with either 25 µL of either DMEM-F12 (5% BSA, control) or 25 µL of a 100 µM PMPA (2-(Phosphonomethyl)-pentandioic acid, Tocris Bioscience, Bristol, UK) solution in PBS, for blockade. Next, 25 µL of the radioactive-labelled PSMA inhibitor (5.0 nM in PBS) was added and the cells were incubated at 37 °C for 60 min. The experiment was terminated by placing the 24-well plate on ice for 3 min and consecutive removal of the medium. Each well was carefully washed with 250 µL of ice-cold HBSS. Both fractions from the first steps, representing the amount of free radioligand, were combined. Removal of surface bound activity was accomplished by incubation of the cells with 250 µL of ice-cold PMPA (10 µM in PBS) solution for 5 min

and rinsed again with another 250 µL of ice-cold PBS. The internalized activity was determined by incubation of the cells in 250 µL 1 M aqueous NaOH for at least 10 min. The obtained fractions were combined with those of the subsequent wash step with 250 µL 1 M aqueous NaOH. Each experiment (control and blockade) was performed in triplicate. Free, surface bound and internalized activity was quantified in a γ-counter. All internalization studies were accompanied by external reference studies, using (^{125}I-I-BA)KuE (0.2 nM/well), which were performed analogously. Data were corrected for non-specific binding and normalized to the specific-internalization observed for the radioiodinated reference compound.

8. Supplemental Data 1

Supplemental Table 1. Binding affinities (IC_{50} in nM, 1 h, 4°C; n=3) of ^{nat}Ga-^{19}F-rhPSMA-5–10, ^{19}F-rhPSMA-5–10 with free chelator, ^{19}F-DCFPyL and ^{19}F-PSMA-1007; internalized activity of ^{18}F-DCFPyL, ^{18}F-PSMA-1007, ^{68}Ga-^{19}F-rhPSMA-5–10 and ^{18}F-rhPSMA-5–10 with free chelator in LNCaP cells (1 h, 37°C) as percent of the reference ligand (^{125}I-I-BA)KuE; n=3); lipophilicity of ^{18}F-DCFPyL, ^{18}F-PSMA-1007, ^{68}Ga-^{19}F-rhPSMA-5–10 and ^{18}F-rhPSMA-5–10 with free chelator, expressed as octanol/PBS (pH 7.4) partition-coefficient (log $P_{oct/PBS}$; n=6); HSA binding of ^{19}F-DCFPyL, ^{19}F-PSMA-1007, ^{nat}Ga-^{19}F-rhPSMA-5–10, determined on a Chiralpak HSA column. Data of reference ligands $^{18/19}$F-DCFPyL and $^{18/19}$F-PSMA-1007 from a previously published study (*3*). Values are expressed as mean ± standard deviation.

Ligand	IC_{50} [nM]	Internalization [% of reference]	Lipophilicity log $P_{O/PBS}$	HSA-binding [%]
$^{68/nat}$Ga-$^{19/18}$F-rhPSMA-5	10.8 ± 2.5	43 ± 3	- 3.0 ± 0.09	-
$^{19/18}$F-rhPSMA-5	8.5 ± 1.7	-	-	
$^{68/nat}$Ga-$^{19/18}$F-rhPSMA-6	7.3 ± 0.2	33 ± 2	- 2.8 ± 0.07	-
$^{19/18}$F-rhPSMA-6	6.4 ± 0.2	-	-	
$^{68/nat}$Ga-$^{19/18}$F-rhPSMA-7	3.0 ± 0.7	126 ± 13	- 3.2 ± 0.05	96
$^{19/18}$F-rhPSMA-7	3.5 ± 0.2	165 ± 5	- 2.0 ± 0.04	-
$^{68/nat}$Ga-$^{19/18}$F-rhPSMA-8	3.8 ± 0.7	98 ± 12	- 2.6 ± 0.04	97
$^{19/18}$F-rhPSMA-8	2.5 ± 0.2	130 ± 6	- 2.3 ± 0.07	-
$^{68/nat}$Ga-$^{19/18}$F-rhPSMA-9	4.5 ± 0.3	180 ± 12	- 3.3 ± 0.06	95
$^{19/18}$F-rhPSMA-9	4.3 ± 0.2	212 ± 5	- 2.2 ± 0.07	-
$^{68/nat}$Ga-^{19}F-rhPSMA-10	3.8 ± 0.3	131 ± 14	- 3.5 ± 0.07	94
$^{19/18}$F-DCFPyL	12.3 ± 1.2	118 ± 4	- 3.4 ± 0.03	14
$^{19/18}$F-PSMA-1007	4.2 ± 0.5	118 ± 5	- 1.6 ± 0.02	98

9. Supplemental Data 2

Supplemental Table 2. Biodistribution of ^{68}Ga-^{19}F-rhPSMA-7 to 10, ^{18}F-rhPSMA-7 and the reference ligands ^{18}F-DCFPyL and ^{18}F-PSMA-1007 at 1 h p.i. in LNCaP tumor-bearing SCID mice (n=3 for ^{68}Ga-^{19}F-rhPSMA-7 to 9, n=3 for ^{18}F-rhPSMA-7, n=4 for ^{68}Ga-^{19}F-rhPSMA-10, ^{18}F-DCFPyL and ^{18}F-PSMA-1007). Data for reference ligands were taken from a previously published study by our group (*3*). Values are expressed as a percentage of injected dose per gram (%ID/g), mean ± standard deviation.

Organ	^{18}F-DCFPyL	^{18}F-PSMA-1007	^{68}Ga-^{nat}F-rhPSMA-7	^{68}Ga-^{nat}F-rhPSMA-8	^{68}Ga-^{nat}F-rhPSMA-9	^{68}Ga-^{nat}F-rhPSMA-10	^{18}F-rhPSMA-7 (uncomplexed)
**Blood**	0.34 ± 0.30	0.48 ± 0.29	1.9 ± 0.1	1.7 ± 0.1	0.46 ± 0.11	1.5 ± 0.3	1.9 ± 0.8
**Heart**	0.25 ± 0.09	1.4 ± 0.5	1.2 ± 0.3	0.93 ± 0.08	0.48 ± 0.17	0.75 ± 0.13	1.0 ± 0.4
**Lung**	0.46 ± 0.05	1.8 ± 1.0	1.7 ± 0.2	1.3 ± 0.2	0.94 ± 0.20	1.3 ± 0.2	2.0 ± 0.7
**Liver**	1.7 ± 0.2	1.2 ± 0.5	0.77 ± 0.10	0.79 ± 0.17	0.42 ± 0.07	0.70 ± 0.20	0.91 ± 0.21
**Spleen**	9.6 ± 4.4	16.68 ± 5.09	13.8 ± 5.0	6.3 ± 1.5	25.4 ± 5.2	9.4 ± 1.6	11.0 ± 2.9
**Pancreas**	0.20 ± 0.10	1.4 ± 0.7	0.71 ± 0.11	0.48 ± 0.11	0.39 ± 0.09	0.51 ± 0.03	0.55 ± 0.18
**Stomach**	0.20 ± 0.03	0.96 ± 0.86	0.51 ± 0.07	0.42 ± 0.11	0.48 ± 0.40	0.42 ± 0.12	0.66 ± 0.18
**Intestine**	0.14 ± 0.02	1.1 ± 0.3	0.43 ± 0.07	0.35 ± 0.01	0.44 ± 0.34	0.47 ± 0.21	0.67 ± 0.23
**Kidneys**	152.4 ± 15.3	123.3 ± 52.1	34.1 ± 6.6	82.3 ± 5.9	54.9 ± 3.0	77.5 ± 13.6	71.9 ± 6.8
**Adrenals**	5.0 ± 4.0	7.5 ± 3.4	6.7 ± 2.2	2.2 ± 0.8	2.7 ± 0.2	2.2 ± 0.3	14.6 ± 7.0
**Muscle**	0.11 ± 0.02	0.64 ± 0.41	0.42 ± 0.22	0.24 ± 0.02	0.19 ± 0.06	0.33 ± 0.10	0.37 ± 0.14
**Bone**	0.12 ± 0.02	0.89 ± 0.67	0.52 ± 0.11	0.32 ± 0.07	0.18 ± 0.05	0.35 ± 0.13	0.70 ± 0.14
**Tumor**	7.3 ± 1.5	7.4 ± 0.7	8.6 ± 0.2	6.5 ± 0.8	7.2 ± 0.6	11.7 ± 1.9	5.5 ± 0.6

10. Supplemental Data 3

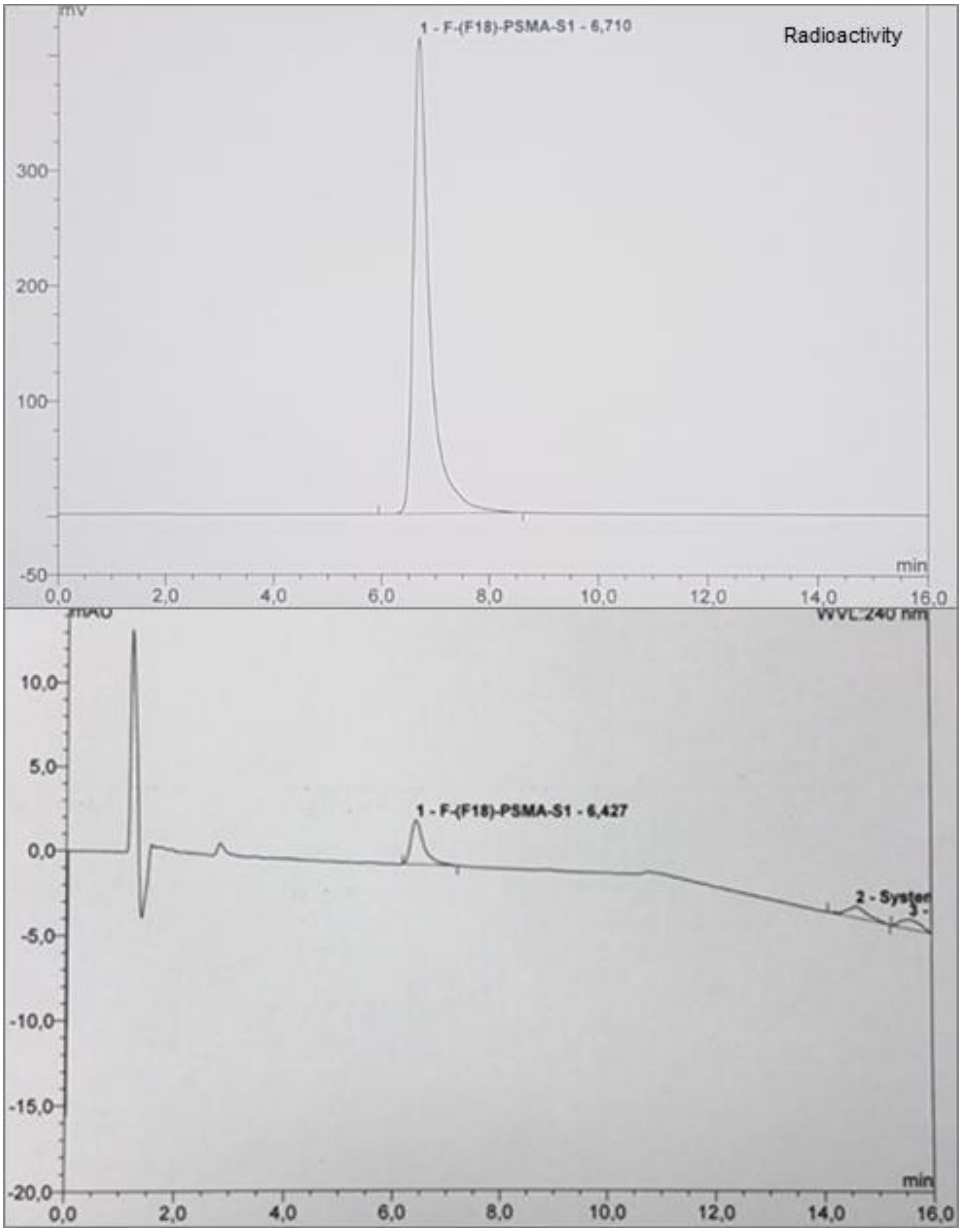

Supplemental Figure 13. Exemplary radio-HPLC analysis of ^{18}F-labelled rhPSMA-7 performed on a Prominence system, equipped with a variable wavelength detector (both Shimadzu) and a gamma-detector Gabi Star (Elysia-raytest, Straubenhardt, Germany). Water/0.1% TFA (solvent A) and MeCN/0.1% TFA (solvent B) served as mobile phases, a Nucleosil 100-5 C18 column of 125 × 4 mm was used as stationary

phase. 10 µl of product solution were injected and the following linear solvent gradient was applied: 30-38 % B in 9 min, 38-95 % B in 8 min, back to 30% B in 1 min and re-equilibration at 30% B for 1.5 min (flow rate = 1 ml/min). Chemical impurities were monitored at 240 nm, the column temperature was set to 30 °C. The system was controlled by Chromeleon 6.8 Chromatography Data System Software (Thermo Fischer Scientific).

10. References

1. Weineisen M, Simecek J, Schottelius M, Schwaiger M, Wester HJ. Synthesis and preclinical evaluation of DOTAGA-conjugated PSMA ligands for functional imaging and endoradiotherapy of prostate cancer. *EJNMMI Res.* 2014;4:63.

2. Weineisen M, Schottelius M, Simecek J, et al. ^{68}Ga- and ^{177}Lu-Labeled PSMA I&T: Optimization of a PSMA-Targeted Theranostic Concept and First Proof-of-Concept Human Studies. *J Nucl Med.* 2015;56:1169-1176.

3. Robu S, Schmidt A, Eiber M, et al. Synthesis and preclinical evaluation of novel ^{18}F-labeled Glu-urea-Glu-based PSMA inhibitors for prostate cancer imaging: a comparison with ^{18}F-DCFPyl and ^{18}F-PSMA-1007. *EJNMMI Res.* 2018;8:30.

4. Iovkova L, Wängler B, Schirrmacher E, et al. para-Functionalized Aryl-di-tert-butylfluorosilanes as Potential Labeling Synthons for ^{18}F Radiopharmaceuticals. *Chem Eur J.* 2009;15:2140-2147.

5. Reich D, Wurzer A, Wirtz M, et al. Dendritic poly-chelator frameworks for multimeric bioconjugation. *Chem Commun.* 2017;53:2586-2589.

6. Notni J, Pohle K, Wester HJ. Comparative gallium-68 labeling of TRAP-, NOTA-, and DOTA-peptides: practical consequences for the future of gallium-68-PET. *EJNMMI Res.* 2012;2:28.

7. Valko K, Nunhuck S, Bevan C, Abraham MH, Reynolds DP. Fast gradient HPLC method to determine compounds binding to human serum albumin. Relationships with octanol/water and immobilized artificial membrane lipophilicity. *J Pharm Sci.* 2003;92:2236-2248.

8. Yamazaki K, Kanaoka M. Computational prediction of the plasma protein-binding percent of diverse pharmaceutical compounds. *J Pharm Sci.* 2004;93:1480-1494.
